# Deletion of Cd248 in *Postn*^+^ myofibroblast fails to attenuate pressure-overload induced cardiac remodeling and fibrosis in mice

**DOI:** 10.1016/j.jmccpl.2026.100837

**Published:** 2026-03-04

**Authors:** Donghua Li, Dongmei Zhong, Tian Tan, Zhilei Huang, Mingyue Wu, Yongshan Liu, Yalin Zhang, Chen Liu, Jie Xu, Fu-Li Xiang

**Affiliations:** a Department of Cardiology, the First Affiliated Hospital of Sun Yat-sen University, Guangzhou, China; b Institute of Precision Medicine, the First Affiliated Hospital of Sun Yat-sen University, Guangzhou, China; c Department of Anesthesia, the First Affiliated Hospital of Sun Yat-sen University, Guangzhou, China; d NHC Key Laboratory of Assisted Circulation and Vascular Diseases, Sun Yat-sen University, Guangzhou, China; e National-Guangdong Joint Engineering Laboratory for Diagnosis and Treatment of Vascular Diseases, Guangzhou, China

**Keywords:** Cd248, Periostin, Myofibroblast, Cardiac fibrosis, Pressure overload, Transverse aortic constriction (TAC)

## Abstract

Cardiac fibrosis driven by activated myofibroblasts is a central feature of pressure-overload heart disease. CD248 (Endosialin/TEM1) has been implicated as a pro-fibrotic marker in ischemic cardiac injury, but its role in pressure overload remains unclear. Here, we tested whether selective deletion of Cd248 in Periostin-expressing (*Postn*^*+*^) myofibroblasts mitigates pressure-overload cardiomyopathy induced by transverse aortic constriction (TAC). We generated inducible PostnMCM^+/−^;Cd248^fl/fl^ mice and validated tamoxifen-driven recombination specifically in *Postn*^*+*^ interstitial cells. *In vitro*, Cd248 knockdown in primary adult mouse cardiac fibroblasts reduced TGF-β1–induced migration and the expression of actomyosin contractile markers; however, it notably failed to suppress the expression of key matrix genes (*Col1a1*, *Postn*), indicating a molecular uncoupling of cytoskeletal dynamics from collagen synthesis. Consistent with this, Postn-restricted Cd248 deletion in vivo produced no significant differences in survival, echocardiographic indices, histological fibrosis, or cardiomyocyte hypertrophy compared to controls at 8 weeks post-TAC. Furthermore, while *Cd248*^*+*^ fibroblasts in the TAC model were enriched for chemotactic signaling programs, immune cell infiltration remained negligible, rendering this immunomodulatory function redundant. These data indicate that while CD248 regulates fibroblast cytoskeletal dynamics *in vitro*, it is dispensable for matrix production and fibrosis in vivo during pressure overload. Our results highlight the profound context-dependence of CD248 as an anti-fibrotic target.

**eTOC blurb:**

In pressure‑overloaded mouse hearts, deleting *Cd248* specifically in *Postn*^+^ myofibroblasts fails to improve cardiac remodeling or fibrosis, despite clear cell-autonomous effects on cultured primary cardiac fibroblast migration and activation *in vitro*. Single‑cell and transcriptomic analyses reveal that while CD248 regulates actomyosin-related cytoskeletal dynamics, it is dispensable for extracellular matrix production in this specific hemodynamic context. These findings highlight the profound context‑dependent efficacy of CD248‑targeted antifibrotic strategies across different etiologies of heart disease.

## Highlights

• Cd248 deletion in *Postn*^+^ cells fails to blunt pressure-overload induced fibrosis despite regulating cytoskeletal dynamics.

## Graphical abstract

**Figure:**
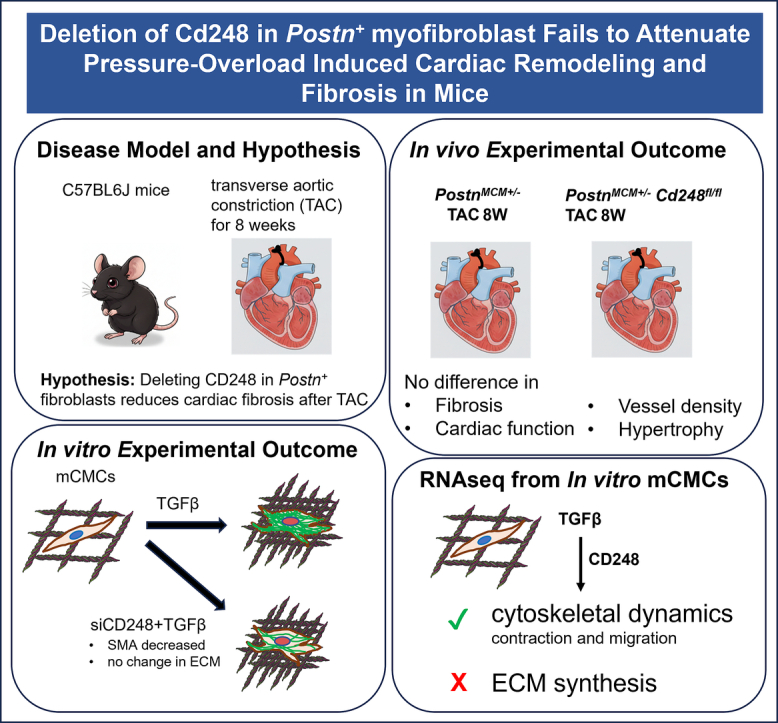

## 1 Introduction

Heart failure, a leading cause of mortality worldwide, often results from maladaptive cardiac remodeling in response to chronic injury stimuli such as pressure overload from hypertension or aortic stenosis [1]. A central pathological hallmark of this process is cardiac fibrosis, characterized by the excessive deposition of extracellular matrix (ECM) proteins [2]. While initially a reparative response, sustained fibrosis leads to increased myocardial stiffness, diastolic dysfunction, and arrhythmogenic substrates, ultimately driving the progression to heart failure. Therefore, elucidating the cellular and molecular drivers of fibrosis is urgently needed for developing effective new therapies.

The resident cardiac fibroblast (CF) has emerged as the principal architect of both adaptive and maladaptive cardiac remodeling [2]. Upon injury, quiescent CFs activate and differentiate into myofibroblasts, which are characterized by the expression of proteins such as Periostin (*Postn*) and smooth muscle actin (*SMA*) [3]. As SMA is also strongly expressed in smooth muscle cells, the *Postn*-Cre and *Postn*-MerCreMer drivers have become invaluable and widely used tools for specifically targeting activated CF population in vivo [2], [4], [5]. *Postn*^+^ fibroblast-specific deletion of the transcription factor KLF5 was shown to attenuate not only pressure-overload induced fibrosis but also cardiomyocyte hypertrophy, revealing a critical paracrine cross-talk (via IGF-1) between fibroblasts and muscle cells [6]. Similarly, our own work and that of others have demonstrated that targeting the canonical WNT/β-catenin or TGF-β-Smad3 signaling pathway specifically within the *Postn*^+^ fibroblast lineage is sufficient to robustly block the fibrotic response to pressure overload [3], [7]. Moreover, the specific ablation of the collagen chaperone Hsp47 in *Postn*^+^ fibroblasts abrogated pressure-overload induced fibrosis and blunted the hypertrophic response, confirming *Postn*^+^ CFs as the primary source of pathological collagen [8]. These studies solidify *Postn*^+^ CFs as a central and validated target for anti-fibrotic therapies.

CD248 is a type-I transmembrane glycoprotein with C-type lectin and EGF-like domains that is expressed at low levels in most adult tissues but is induced on mesenchymal stromal cells, notably pericytes, vascular smooth muscle cells (VSMCs) and fibroblasts, during development, inflammation and remodeling [9], [10]. Genetic and biochemical studies show that CD248 participates in vascular patterning and plays a modulatory role in microvascular remodeling [11]. CD248 promotes VSMC remodeling and plaque development in atherosclerosis [12]. Recently, two parallel studies using single-cell transcriptomics discovered that CD248 (Endosialin/TEM1) is a specific surface marker for a late-activated, pathological fibroblast subpopulation that drives chronic fibrosis after myocardial infarction (MI) or ischemia/reperfusion (I/R) [13], [14]. Critically, both studies demonstrated that therapeutic interventions targeting these Cd248^+^ cells, using either CAR-T cell therapy or neutralizing antibodies, successfully ameliorated cardiac fibrosis and improved heart function. One of these studies further showed that deleting *Cd248* specifically in the *Postn* + lineage was sufficient to confer this protection in an I/R model [14].

However, whether CD248 is required for fibrosis in pressure-overload cardiomyopathy is unknown. Here, motivated by an siRNA screen in primary cardiac fibroblasts that highlighted Cd248 as a regulator of TGF-β driven activation, we tested the hypothesis that selective deletion of Cd248 in *Postn*^*+*^ myofibroblasts improves pathological remodeling in pressure-overload cardiomyopathy induced by transverse aortic constriction (TAC). Contrary to expectation, myofibroblast-restricted Cd248 deletion did not reduce TAC-induced fibrosis or pathological remodeling, despite clear cell-autonomous effects of CD248 loss on mesenchymal cell activation, migration, and proliferation in vitro. These findings suggest that CD248's role in cardiac fibrosis is context-dependent, differing across injury types and disease stages, and they emphasize the need to match fibroblast-targeted therapies to the appropriate pathological niche.

## 2 Methods

### 2.1 Animals and husbandry

All animal experiments were approved by the Sun Yat-sen University Animal Care and Use Committee (SYSU-IACUC-2024-002343). Myofibroblast-specific inducible *Cd248* knockout mice (*PostnMCM*^*+/−*^*;Cd248*^*fl/fl*^) and their corresponding controls (*PostnMCM*^*+/−*^, The Jackson Laboratory, 029645) were generated on a C57BL/6 J background. To validate Cre recombinase activity, *PostnMCM*^*+/−*^ mice were crossed with a tdTomato reporter line containing a Loxp-Stop-LoxP cassette. All mice were housed in a specific pathogen-free (SPF) facility on a 12-h light/dark cycle with ad libitum access to food and water. All surgical and experimental procedures were performed on male mice aged 10–14 weeks.

### 2.2 Experimental design and SAGER guidelines

In accordance with SAGER (Sex and Gender Equity in Research) guidelines, we report that this study was conducted exclusively in male mice aged 10–14 weeks. This single-sex design was chosen to minimize biological variability associated with the estrous cycle in females and to avoid the known confounding cardioprotective effects of female sex hormones in cardiac injury models, thereby reducing the total number of animals required to achieve statistical power. We acknowledge that this approach limits the direct generalizability of our findings. The role of CD248 in pressure-overload cardiomyopathy may differ in females, and future studies are warranted to investigate potential sex-specific mechanisms.

For comparisons between genotypes, littermate controls were used whenever possible. Within each genotype, mice were randomly allocated to either the TAC or sham surgery group. Key outcome assessments, including the analysis of echocardiographic data and the quantification of histological fibrosis and cardiomyocyte size, were performed by investigators who were blinded to the genotype and surgical group of the animals.

### 2.3 Statistical analysis

All quantitative data are presented as mean ± SEM. Statistical analyses were performed using GraphPad Prism software (v9.0, GraphPad Software). For comparisons between two groups with normally distributed data, an unpaired, two-tailed Student's *t*-test was used. For comparisons between more than two groups with normally distributed data, a one-way or two-way Analysis of Variance (ANOVA) was performed, followed by Tukey's multiple comparisons post-hoc test. For data that were not normally distributed, the non-parametric Mann-Whitney *U* test (for two groups) or the Kruskal-Wallis test with Dunn's multiple comparisons test (for multiple groups) was used. Survival curves were generated using the Kaplan-Meier method and compared using the Log-rank (Mantel-Cox) test. A *P*-value <0.05 was considered statistically significant. The specific tests used for each experiment are detailed in figure legends.

Please see detailed methodology in Supplemental methods.

## 3 Results

### 3.1 Single-cell analysis reveals Cd248 expression in activated cardiac fibroblasts

**Fig. 1:**
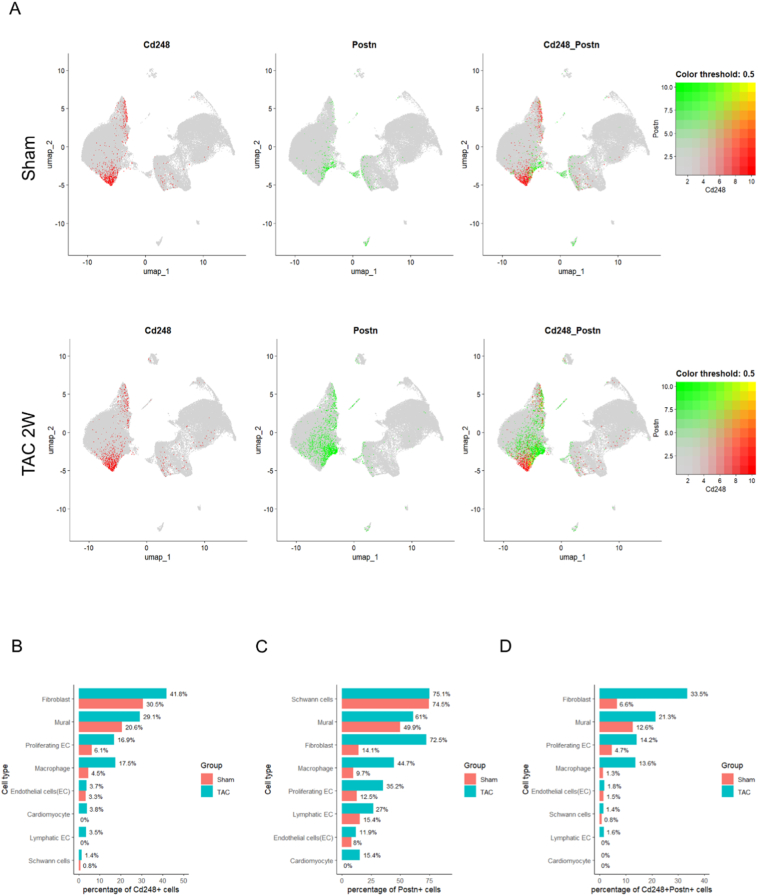
Single-cell RNAseq reveals co-expression of Cd248 and Postn in cardiac fibroblasts and mural cells following pressure overload. (A) Uniform Manifold Approximation and Projection (UMAP) of cardiac interstitial cells from sham and TAC-operated mice, annotated by cell type and experimental group. (B–D) Quantification of the percentage of cells positive for Cd248, Postn, or both within each major cell lineage. Data are presented as percentages for each group (salmon = Sham, teal = TAC). (For interpretation of the references to colour in this figure legend, the reader is referred to the web version of this article.)

To confirm CD248 expression in fibroblasts in pressure overload disease setting, we analyzed published mouse and human single-cell RNA-sequencing datasets. In a public dataset from mouse hearts subjected to TAC (GSE166403) [15], the *Cd248*-positive cell population expanded (Fig. 1A and B), with expression predominantly in fibroblasts, followed by mural cells and endothelial cells (Fig. 1B). Within the fibroblast population 14 days after TAC, the number of *Postn*^+^ cells increased dramatically (Fig. 1A and C), and the majority of *Cd248*^+^ cells co-localized with *Postn* resulting in about 33% of the total fibroblasts being *Postn*^+^*Cd248*^+^ (Fig. 1D). In a published human hypertrophic cardiomyopathy single nuclear RNAseq dataset [16], CD248 was found to be significantly increased and majorly expressed by pericytes, fibroblasts and smooth muscle cells in hypertrophic human heart (Supplemental Fig. 1 A). This confirms our genetic strategy targets the intended pathological *Postn*^+^*Cd248*^+^ myofibroblast population.

We also validated *Cd248* gene expression in various organs and different cardiac injury models. We found that the gene expression of Cd248 is relatively high in heart, lung, kidney, skeletal muscle and brain (Supplemental Fig. 1A). In TAC model, cardiac expression of Cd248 was significantly upregulated 1 week after TAC (Supplemental Fig. 2A). The fibrosis marker (*Col1a1, Col3a1, Postn*, and *Acta2*) and mechano-stress associated membrane frangibility regulator *Ninj1* (Supplemental Fig. 2B) also significantly increased. Moreover, consistent with published studies [13], [14], upregulation of CD248 was also observed in the infarct border zone 3 days after MI (Supplemental Fig. 2C).

### 3.2 Cd248 knockdown attenuates cardiac fibroblast activation, migration, and proliferation *in vitro*

To first establish the functional role of Cd248 in cardiac fibroblasts, we performed knockdown experiments in primary adult mouse cardiac fibroblasts (mCFs). To ensure that our *in vitro* findings were specific to the cardiac fibroblast lineage, we validated the purity of our primary cell cultures, confirming that 100% of cells stained positive for Vimentin (Supplemental Fig. 3A), and RNA-sequencing analysis further corroborated this by demonstrating high expression of fibroblast markers (*Vim*, *Dcn*, *Col1a1*) alongside negligible expression of endothelial, immune, smooth muscle, or cardiomyocyte lineage genes (Supplemental Fig. 3B).

**Fig. 2:**
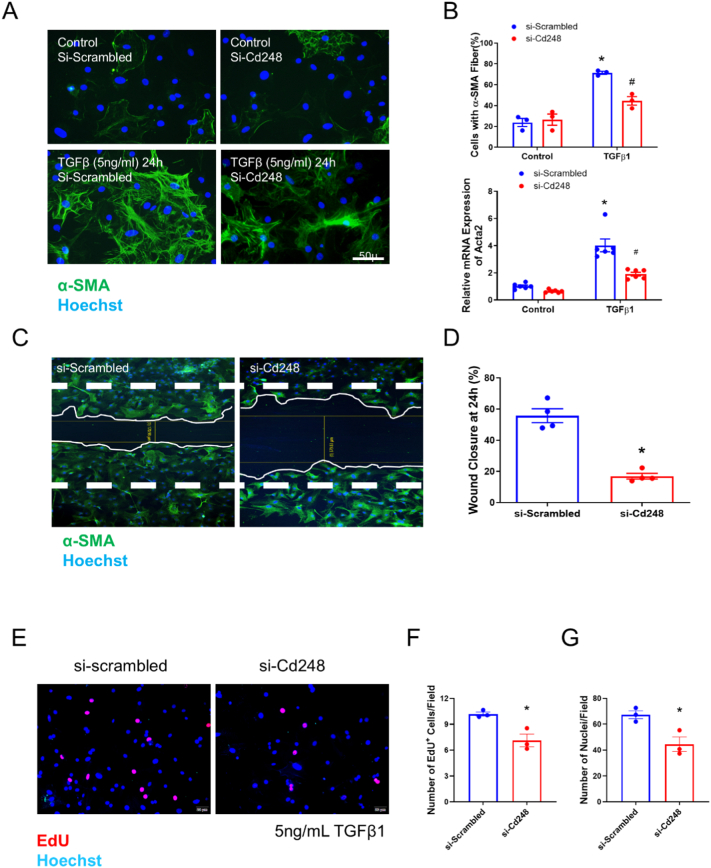
Knockdown of Cd248 blunts TGF-β1-induced fibroblast activation, migration, and proliferation in vitro. (A) Representative immunofluorescence of cultured cardiac fibroblasts transfected with control siRNA (si-Scrambled) or siRNA targeting Cd248 (si-Cd248) and stimulated with vehicle or TGF-β1 (5 ng/mL) for 24 h. Myofibroblast activation is indicated by the formation of α-SMA stress fibers (green); nuclei are counterstained with Hoechst (blue). Scale bar, 50 μm. (B) Quantification of myofibroblast activation, showing the percentage of cells with organized α-SMA stress fibers and the relative mRNA expression of Acta2 (α-SMA). Two-way Analysis of Variance (ANOVA) was performed, followed by Tukey's multiple comparisons post-hoc test. *P < 0.05 vs. the corresponding vehicle control; #*P* < 0.05 vs. si-Scrambled with TGF-β1 stimulation. (D) Scratch-wound assay demonstrating impaired fibroblast migration following Cd248 knockdown. Quantification shows the percentage of wound closure at 24 h relative to the initial scratch. *P < 0.05 vs.si-Scrambled, determined by an unpaired Student's *t-test*. (*E*-G) Analysis of fibroblast proliferation via EdU incorporation. Representative images (E) and quantification of the number of EdU^+^ nuclei (F) and total nuclei (G) per field. *P < 0.05 vs.si-Scrambled, determined by an unpaired Student's *t-test*. *n* = 3–6, data are presented as mean ± SEM; each dot represents an independent experiment. (For interpretation of the references to colour in this figure legend, the reader is referred to the web version of this article.)

Pathological stimulation with TGF-β1 robustly induced mCFs activation into myofibroblasts, characterized by a ∼ 4-fold increase in *Acta2* (α-SMA) mRNA and the formation of prominent α-SMA stress fibers (Fig. 2A and B). Knockdown of *Cd248* (Supplemental Fig. 4A) significantly inhibited this activation process (Fig. 2A and B). Migration was measured by wound scratch assay. Knocking down of *Cd248* in mCFs exhibited significantly slower wound healing compared to the control group (Fig. 2C and D). Moreover, cell proliferation was detected by EdU staining. Both the total cell number and the number of proliferating cells marked by EdU were significantly lower in the TGF-β1 treated *Cd248* knockdown mCFs (Fig. 2E-G). *Cd248* knockdown mCFs showed less *Col3a1* gene expression, but no decreases in *Col1a1* and *Postn* (Supplemental Fig. 4B–D). We further validated the efficiency of our knockdown strategy using immunofluorescence staining, which confirmed the specific ablation of CD248 protein in si-Cd248 treated cells while the protein expression of the myofibroblast marker Periostin remained unaffected (Supplemental Fig. 4E). To rule out potential off-target effects, we repeated these key *in vitro* experiments using a second, independent set of siRNAs targeting Cd248 (Supplemental Fig. 5A), which yielded virtually identical results: significant suppression of TGF-β1-induced α-SMA stress fiber formation and *Acta2* expression without altering *Postn* levels (Supplemental Fig. 5B–C). This data suggest that *Cd248* might play an important role in mCFs activation, migration and proliferation.

### 3.3 *Postn*^*+*^ myofibroblast-specific deletion of Cd248 has no impact on cardiac fibrosis or adverse remodeling in mouse TAC model

Given the pro-fibrotic function of Cd248 in vitro, we tested if deleting it in *Postn*^+^ myofibroblasts would protect against TAC-induced pathology. We validated the successful establishment of TAC model in-house. In all TAC mice in this study, pulsed-wave Doppler echocardiography was used to confirm successful constriction, with peak blood flow velocity across the aortic arch increasing from a baseline of ∼1000 mm/s to at least 3500 mm/s post-TAC (Supplemental Fig. 6A). Histological analysis via Picro-Sirius Red staining of WT hearts 6 weeks post-TAC confirmed the development of pathological fibrosis, showing a significant increase in both interstitial and perivascular fibrosis (Supplemental Fig. 6B). Finally, it is important to contextualize our findings within the specific physiology of the C57BL/6 J mouse strain used in this study; consistent with the well-documented resistance of this strain to the transition to heart failure [17], our long-term follow-up revealed that Ejection Fraction remained relatively normal even up to 16 weeks post-TAC (Supplemental Fig. 6C), confirming a phenotype of “compensated hypertrophy” despite robust fibrosis (Supplemental Fig. 6B) and significant CD248 upregulation (Supplemental Fig. 6D).

To specifically delete Cd248 in *Postn*^+^ myofibroblasts, we used the *PostnMCM*^*+/−*^*;Cd248*^*fl/fl*^ (Postn-Cd248) and *PostnMCM*^*+/−*^ (control) mouse lines, where tamoxifen induces Cre expression in emerging *Postn*^+^ cells. We rigorously validated our genetic model, confirming that the *Postn*-MCM driver targeted interstitial cells without labeling cardiomyocytes (Supplemental Fig. 7A) and efficiently deleted CD248 in Postn^+^ myofibroblasts (Supplemental Fig. 7B); crucially, CD248 expression was preserved in CD31^+^ endothelial cells in the knockout hearts (Supplemental Fig. 7C), corroborating our single-cell observation that non-fibroblast lineages constitute a significant and unaffected source of CD248 in the pressure-overloaded heart. This histological validation robustly confirms both the efficiency and specificity of the deletion of the target gene in our genetic mouse system. The Cre recombination efficiency is comparable to the previous study using the same *Postn*-MCM system generated by Dr. Molkentin [3], [7].

**Fig. 3:**
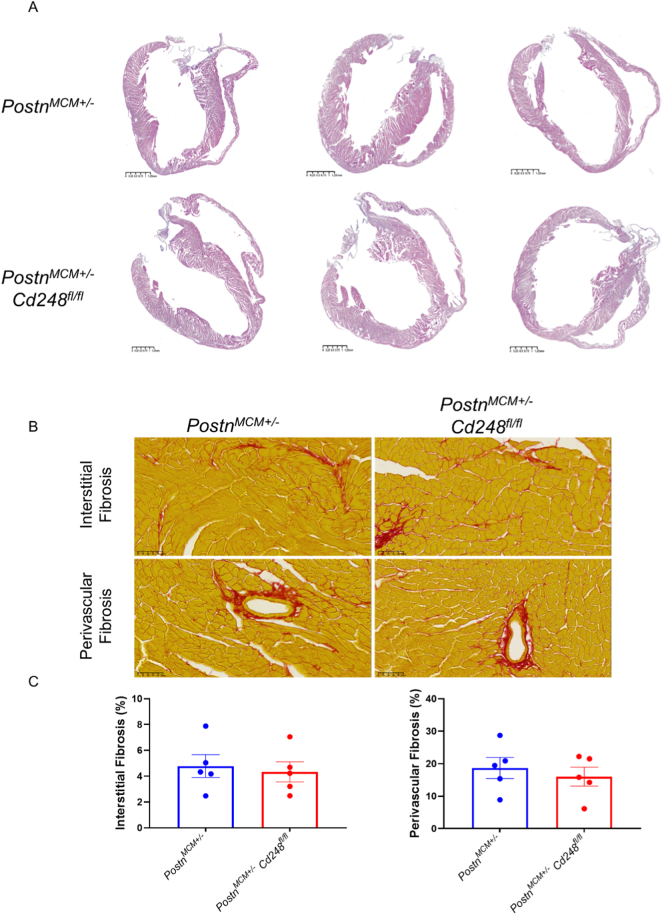
*Postn*^+^ myofibroblast-specific deletion of Cd248 does not alter cardiac fibrosis after pressure overload. (A) Representative low-magnification images of HE-stained long-axis heart sections 8 weeks after transverse aortic constriction (TAC). Scale bar, 1.25 mm. (B) High-magnification images of Picro-Sirius Red-stained heart section of control (Postn-MCM) and *Postn*^+^myofibroblast-specific Cd248 knockout (Cd248-cKO) mice are shown. Collagen is stained red, and myocardium appears yellow. Detailing interstitial fibrosis (top) and perivascular fibrosis (bottom) for each genotype were shown. Scale bar, 50 μm. (C) Quantification of the fibrotic area. Data are presented as mean ± SEM; *n* = 5, each dot represents an individual animal. Statistical analysis was performed as described in the Methods. (For interpretation of the references to colour in this figure legend, the reader is referred to the web version of this article.)

**Fig. 4:**
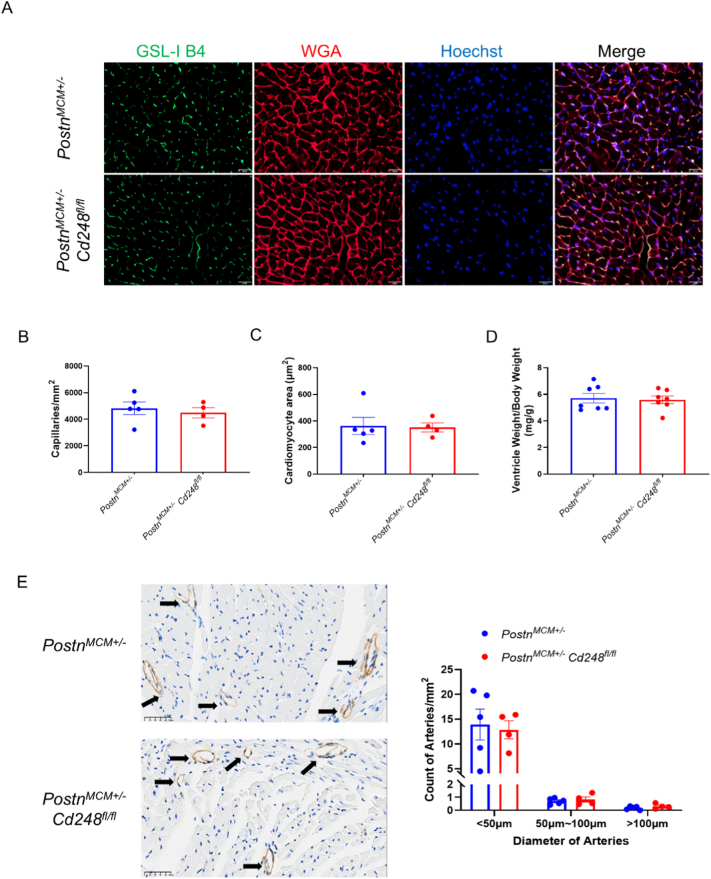
*Postn*^+^ myofibroblast-specific deletion of Cd248 does not alter cardiomyocyte hypertrophy or microvascular density in response to pressure overload. (A) Representative confocal images of left ventricular cross-sections from control and Cd248-cKO mice 8 weeks after TAC surgery. Capillaries are labeled with Isolectin-B4 (IB4, green), cardiomyocyte membranes with Wheat Germ Agglutinin (WGA, red), and nuclei with DAPI (blue). Scale bar, 50 μm. (B–D) Quantification of morphometric parameters. No significant differences were observed between control (blue bars) and Cd248-cKO (red bars) mice in (B) capillary density, (C) cardiomyocyte cross-sectional area, or (D) the ventricle weight to body weight ratio. (E) Representative images of arterioles identified by α-smooth muscle actin (α-SMA) immunohistochemistry (arrows). Scale bar, 50 μm. Quantification of α-SMA^+^ arterioles, showing no significant differences in total arteriolar density (left) or in the distribution of arteriolar sizes (right) between genotypes. Data are presented as mean ± SEM; *n* = 4–10, each dot represents an individual animal. Statistical analysis was performed as described in the Methods. (For interpretation of the references to colour in this figure legend, the reader is referred to the web version of this article.)

Eight weeks after TAC, hearts from Postn-Cd248 knockout and control mice were harvested and submitted to comprehensive cardiac function and histology analysis. Peak blood flow velocity across the aortic arch in both groups was confirmed to be similar (Supplemental Fig. 8A). Both groups had survival rates above 80% during the 8 weeks after TAC (Supplemental Fig. 8B). HE staining of the 4-chamber view showed no changes in global morphology (Fig. 3A). Quantification of Picro-Sirius Red-stained heart sections revealed that the fibrotic area, measured as both interstitial and perivascular collagen deposition, was extensive and statistically indistinguishable between the Postn-Cd248 knockout and control mice (Fig. 3B and C). Furthermore, vessel density and myocardial hypertrophy were also analyzed. There were no significant differences in myocardial capillary density, cardiomyocyte cross-sectional area, or ventricular weight to body weight ratio between the two groups (Fig. 4A-D). Cd248 has previously been shown to regulate arteriole remodeling in cancer [11], [18]. Thus, we quantified the density of arterioles with three different size categories: <50 μm, 50-100 μm and > 100 μm. No difference in arteriole densities was detected between the Postn-Cd248 knockout and control mouse hearts (Fig. 4E). Taken together, these histological data unequivocally demonstrate that deleting Cd248 specifically within the *Postn*^*+*^ myofibroblast lineage fails to mitigate the myocardial pathological remodeling of chronic pressure overload.

### 3.4 Myofibroblast-specific deletion of *Cd248* fails to mitigate cardiac dysfunction

**Fig. 5:**
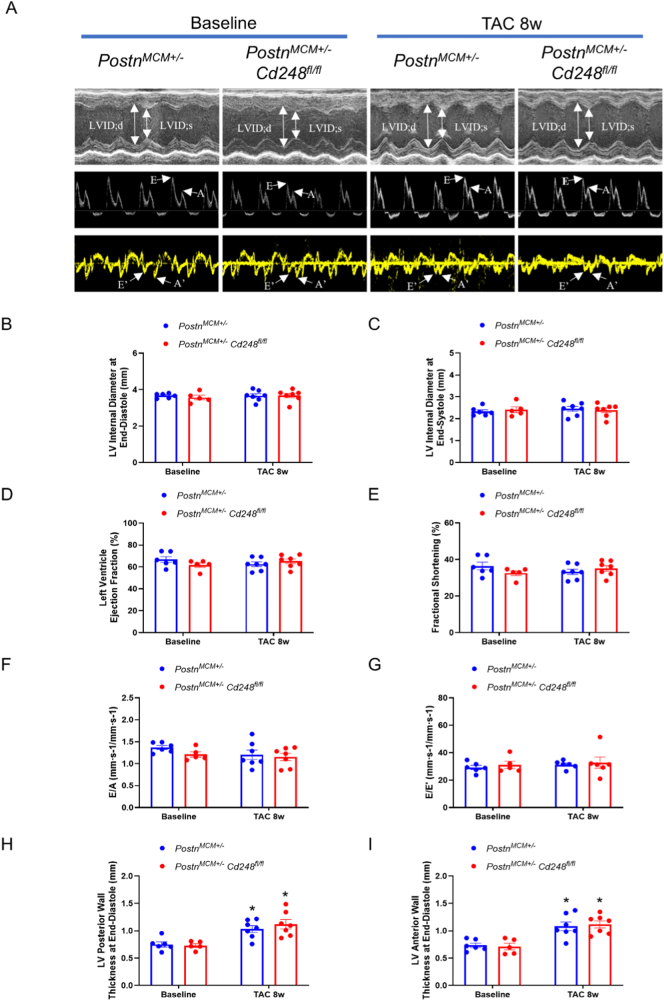
*Postn*^+^ myofibroblast-specific deletion of Cd248 does not alter cardiac function or dimensions after pressure overload. (A) Representative echocardiographic recordings from control and Cd248-cKO mice at baseline and 8 weeks after TAC. Images shown are M-mode, pulsed-wave Doppler of transmitral inflow, and tissue-Doppler imaging (TDI) of the mitral annulus. (B–I) Quantification of key echocardiographic parameters at baseline and 8 weeks post-TAC for control (blue bars) and Cd248-cKO (red bars) mice. Parameters shown include left ventricular internal dimensions at diastole and systole (LVID;d, LVID;s), systolic function (ejection fraction [EF], fractional shortening [FS]), diastolic indices (E/A, E'/A', and E/E') and posterior wall thickness (LVPW;d, LVPW;s). No significant differences were observed between genotypes. Data are presented as mean ± SEM; *n* = 5–7, each dot represents an individual animal. Two-way Analysis of Variance (ANOVA) was performed, followed by Tukey's multiple comparisons post-hoc test. *P < 0.05 vs. baseline. (For interpretation of the references to colour in this figure legend, the reader is referred to the web version of this article.)

To confirm whether there would be changes in cardiac function or the cardiac muscle dynamic, which might be missed in histology, we performed echocardiographic assessment including traditional M-mode, B-mode pulsed-wave doppler, tissue doppler imaging (Fig. 5A) and speckle-tracking based muscle strain analysis in both groups. Consistent with histological findings, there are no changes in FS, EF, LVID, E/A and E/E' at 8 weeks post-TAC between Postn-CD248 knockout and control mice (Fig. 5B-G). Both groups developed significant and comparable concentric hypertrophy (Fig. 5H and I). Speckle-tracking analysis based on strain analysis also revealed no difference between cardiac muscle movement between Postn-CD248 knockout and control mice (Supplemental Fig. 9A-D).

### 3.5 Single-cell analysis reveals distinct transcriptional programs in *Cd248*^*+*^ and *Postn*^*+*^ fibroblast subpopulations

**Fig. 6:**
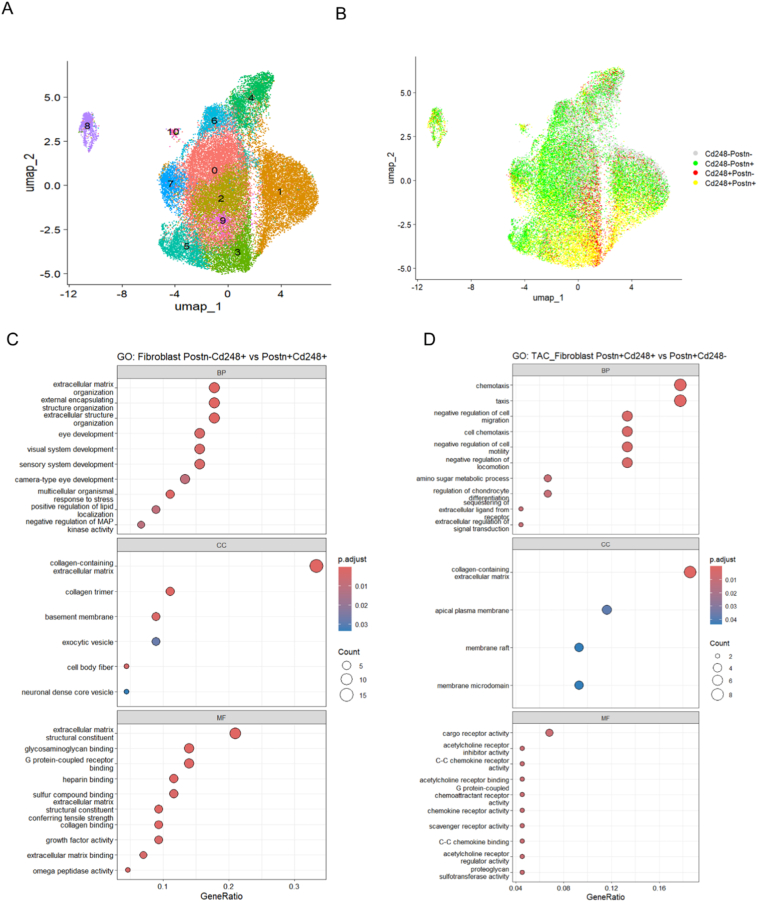
Single-cell transcriptomic analysis reveals distinct biological programs in fibroblast subpopulations from pressure-overloaded hearts. (A) UMAP visualization of cardiac fibroblasts isolated from mice 14 days after TAC, showing unsupervised clustering into distinct subpopulations (0−10). (B) *Cd248*^*−*^*Postn*^*−*^ (grey), *Cd248*^*−*^*Postn*^*+*^ (green), *Cd248*^*+*^*Postn*^*−*^ (red)and their co-expression (yellow) within the fibroblast UMAP. (C–D) Gene Ontology (GO) enrichment analysis of differentially expressed genes (DEGs) from the TAC fibroblast population. Dot plots show the top enriched biological programs comparing (C) *Postn*^*−*^*Cd248*^*+*^ versus *Postn*^*+*^*Cd248*^*+*^ fibroblasts and (D) *Postn*^*+*^*Cd248*^*+*^ versus *Postn*^*+*^*Cd248*^*−*^ fibroblasts. Dot size corresponds to the number of DEGs mapped to each term, and colour indicates the false discovery rate (FDR). (BP, Biological Process; CC, Cellular Component; MF, Molecular Function). (For interpretation of the references to colour in this figure legend, the reader is referred to the web version of this article.)

To understand the potential heterogeneity of the target cell population, we further analyzed the fibroblast cluster from the scRNA-seq data of TAC hearts [15]. Unsupervised clustering resolved the fibroblasts into 11 distinct subpopulations (Fig. 6A). A bivariate feature plot in UMAP revealed four main groups based on the expression of Cd248 and Postn: double-positive (*Cd248*^*+*^*Postn*^*+*^), *Cd248* single-positive, *Postn* single-positive, and double-negative cells (Fig. 6B). To elucidate the functional differences between these subpopulations, we performed differential gene expression analysis followed by Gene Ontology (GO) enrichment. When comparing the double-positive population to the Cd248 single-positive (*Postn*^*−*^*Cd248*^*+*^) population, the enriched terms were related to extracellular matrix/structure organization confirming the importance of *Postn*^*+*^ cells in fibrosis development (Fig. 6C). However, when comparing the double-positive (*Cd248*^*+*^*Postn*^*+*^) population to the *Postn* single-positive (*Postn*^*+*^*Cd248*^*−*^) population (Fig. 6D), we found that within the *Postn*^*+*^ fibroblast population, the CD248 expressing cells were significantly associated with chemotaxis and migration not ECM remodeling. These results suggest that fibroblasts co-expressing *Cd248* and *Postn* represent an immune reaction related subpopulation, while the fibrosis associated effects might be dependent on the *Postn*^*+*^*Cd248*^*−*^ fibroblast subsets.

We identified a panel of signature genes associated with chemotaxis in this *Postn*^*+*^*Cd248*^*+*^ subset, including *Ackr3*, *S100a4*, and *Sema3c*, which were consistently upregulated in this population in vivo (Supplemental Fig. 10A). However, when we knocked down Cd248 in TGF-β1-stimulated fibroblasts in vitro, we observed no significant downregulation of these chemotactic markers; in fact, *Ackr3* expression was paradoxically maintained or slightly increased (Supplemental Fig. 10B). This disconnect suggests that while these immune-related genes serve as markers for the *CD248*^*+*^ population in vivo, they are not directly regulated by CD248 signaling in the context of TGFβ treatment. We also performed immunofluorescence staining for CD45 and CD68 in the TAC hearts, we observed very few infiltrating leukocytes (Supplemental Fig. 11A) or macrophages (Supplemental Fig. 11B) in the myocardium of either genotype, despite validating antibody sensitivity in spleen and liver controls (Supplemental Fig. 11C). Collectively, these data suggest that unlike in MI injury [14], where CD248 mediates critical immune-fibroblast crosstalk, the transcriptome changes in chemotaxis genes observed in the *Postn*^*+*^*Cd248*^*+*^ subset in the TAC model did not result in enhanced immune cell infiltration.

Finally, to rigorously test the ‘target burden’ hypothesis—that the density of *CD248*^*+*^ cells in TAC might be too low to elicit a therapeutic benefit compared to the high prevalence reported in MI literature [14], we performed a direct integrated analysis using the published TAC and MI single-cell datasets(Supplemental Fig. 12). This side-by-side comparison revealed that the prevalence of the pathogenic *Cd248*^*+*^*Postn*^*+*^ myofibroblast subpopulation is higher in the pressure-overloaded heart (35%) compared to the ischemic model (14.9%) where CD248 depletion was therapeutic (Supplemental Fig. 13). This finding definitively rules out low target burden as a cause for the null phenotype, rather, it demonstrates that despite being more abundant in pressure overload, *CD248*^*+*^ myofibroblasts are functionally dispensable for fibrosis in this specific hemodynamic context.

### 3.6 Transcriptomic analysis reveals Cd248 regulates actomyosin organization but not ECM production programs

**Fig. 7:**
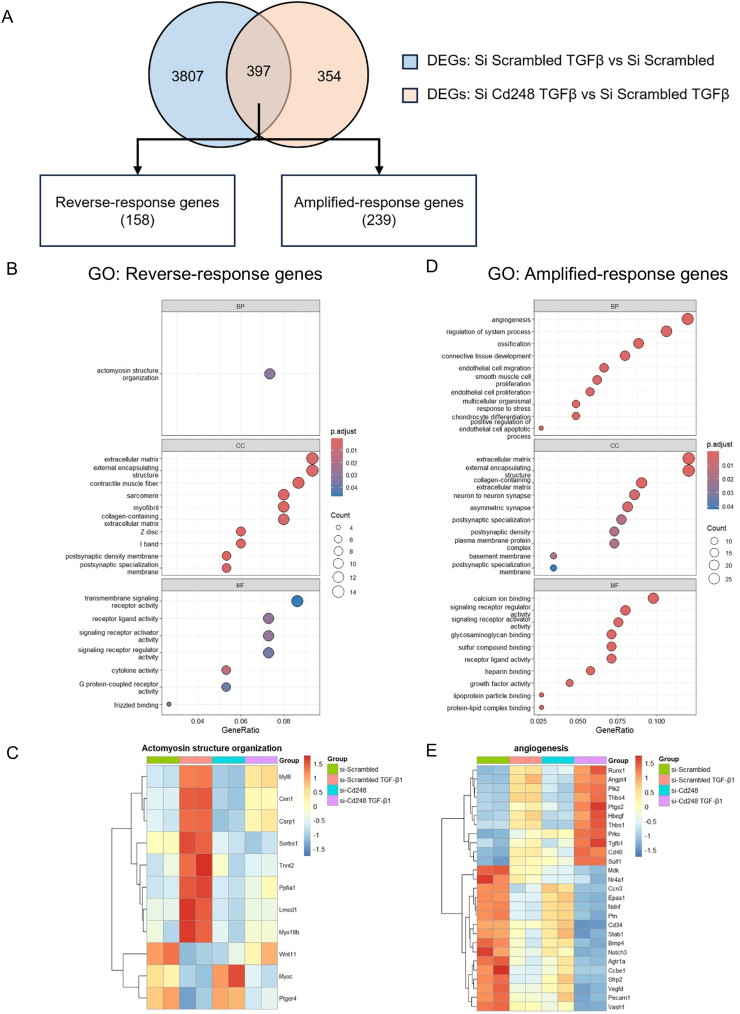
Transcriptomic profiling reveals Cd248 regulates TGF-β1-induced actomyosin organization and angiogenic programs in vitro. (A) Venn diagram and schematic strategy for identifying Cd248-dependent transcriptional changes. Differentially expressed genes (DEGs) induced by TGF-β1 (si-Scrambled TGF-β1 vs. si-Scrambled) were intersected with DEGs modified by Cd248 knockdown (si-Cd248 TGF-β1 vs. si-Scrambled TGF-β1). This analysis identified 158 “Reverse-response genes” (genes where Cd248 knockdown antagonized the TGF-β1 effect) and 239 “Amplified-response genes” (genes where knockdown enhanced the TGF-β1 effect). (B) Gene Ontology (GO) enrichment analysis of the Reverse-response genes. The dot plots display the top enriched terms for Biological Process (BP), Cellular Component (CC), and Molecular Function (MF), highlighting pathways involved in actomyosin structure organization and extracellular matrix. (C) Heatmap of representative Reverse-response genes enriched in the “Actomyosin structure organization” pathway (e.g., Myl9, Cnn1, Wnt11). The colour scale indicates that the TGF-β1-induced upregulation of these genes (red in si-Scrambled TGF-β1) is attenuated by Cd248 knockdown (blue/yellow in si-Cd248 TGF-β1). (D) GO enrichment analysis of the Amplified-response genes. Top enriched BP terms include angiogenesis, endothelial cell migration, and smooth muscle cell proliferation. (E) Heatmap of representative Amplified-response genes enriched in the “Angiogenesis” pathway (e.g., Runx1, Angpt4, Thbs1). The heatmap demonstrates that Cd248 knockdown further potentiates the expression of these genes in the presence of TGF-β1. Colour scale: Red represents high relative expression; Blue represents low relative expression. Values are row-normalized *Z*-scores. (For interpretation of the references to colour in this figure legend, the reader is referred to the web version of this article.)

To elucidate the molecular mechanisms underlying the observed effects of Cd248 knockdown on fibroblast activation and migration, and to investigate why these in vitro effects might not translate to reduced fibrosis in vivo, we performed bulk RNA sequencing on primary mCFs. Cells were transfected with control or Cd248-targeting siRNA in the presence or absence of TGF-β1 stimulation. We identified genes differentially expressed in response to TGF-β1 in control cells and intersected them with genes regulated by Cd248 knockdown. This analysis stratified the transcriptomic changes into two distinct regulatory modules: “Reverse-response genes” (158 genes), where Cd248 knockdown antagonized the TGF-β1 effect, and “Amplified-response genes” (239 genes), where knockdown enhanced the TGF-β1 response (Fig. 7A). Gene Ontology (GO) enrichment analysis of the Reverse-response module revealed a significant enrichment for terms related to contractile apparatus, specifically “actomyosin structure organization,” “sarcomere,” and “myofibril” (Fig. 7B). Heatmap analysis confirmed that the TGF-β1-induced upregulation of key contractile genes—such as *Myl9*, *Cnn1*, *Csrp1*, and *Wnt11*, was markedly blunted in Cd248-knockdown cells (Fig. 7C). Crucially, however, this “Reverse-response” module did not include the primary drivers of collagen deposition. We observed no significant changes in the expression of major extracellular matrix (ECM) genes, such as *Col1a1* and *Postn*, or the broad “collagen-containing extracellular matrix” GO terms within the Reverse-response group. This indicates that while Cd248 is essential for the cytoskeletal organization required for myofibroblast contraction and migration, it is dispensable for the actual synthesis and secretion of the fibrotic matrix. In contrast, the Amplified-response module was enriched for vascular biology terms, including “angiogenesis” and “endothelial cell migration” (Fig. 7D). Cd248 knockdown led to the hyper-induction of angiogenic factors like *Runx1*, *Angpt4*, and *Thbs1* in the presence of TGF-β1 (Fig. 7E). Finally, to test the effects of targeting CD248 in established myofibroblasts, we initiated activation with TGF-β1 for 8 h prior to siRNA transfection; notably, this delayed intervention failed to reverse α-SMA expression or stress fiber formation (Supplemental Fig. 14), suggesting that CD248 is critical for the initial induction rather than the maintenance of the myofibroblast phenotype. Taken together, these data provide a molecular explanation for the lack of an in vivo fibrosis phenotype: the specific deletion of Cd248 impairs the myofibroblast's contractile machinery (affecting migration and potentially stiffness) but fails to halt the massive production of collagen that drives interstitial fibrosis in pressure overload.

## 4 Discussion

The primary finding of this investigation is the absence of an observable cardiac phenotype following the specific deletion of Cd248 in *Postn*-expressing myofibroblasts within a murine model of pressure-overload cardiomyopathy. This result is scientifically significant as it indicates that the pro-fibrotic function of Cd248, which has been well-documented in the context of ischemic cardiac injury, may not be conserved across different etiologies of heart disease. Specifically, it suggests a context-dependent role for Cd248 within the *Postn*^*+*^ myofibroblast lineage.

The rationale for targeting the *Postn*^*+*^ cell lineage was based on extensive prior validation. This approach has been repeatedly validated as an effective strategy to interrogate fibroblast function in the pressure-overloaded heart in our hands as well as in other labs [2], [3], [4], [5], [7], [8]. For example, Takeda et al. used a *Postn-Cre* driver to show that fibroblast-specific deletion of KLF5 attenuates both fibrosis and cardiomyocyte hypertrophy by disrupting paracrine IGF-1 signaling after TAC [6]. Similarly, our group and others have used *Postn-MerCreMer* mice to demonstrate that specifically inhibiting the canonical TGFβ or WNT/β-catenin signaling as well as collagen chaperone Hsp47 in activated fibroblasts robustly blocked pressure-overload induced fibrosis and secondarily attenuated the hypertrophic response [3], [7], [8]. Collectively, these studies confirmed that targeting key molecular pathways within the *Postn*^+^ fibroblast population is a valid and effective strategy for mitigating pressure-overload pathology.

The hypothesis for the present study was further supported by recent findings that identified Cd248 as a specific marker for a late-activated, pathological fibroblast subpopulation in ischemic injury models. Both Chen et al. (2025) and Li et al. (2025) independently showed that therapeutic interventions targeting these *Cd248*^*+*^ cells successfully reduced fibrosis and improved cardiac function after myocardial infarction or ischemia/reperfusion [13], [14]. Our re-analysis of published single-cell sequencing data confirms that *Cd248*^*+*^ fibroblasts constitute a substantial portion (over 30%) of the total fibroblast population in the pressure-overloaded heart, suggesting this subpopulation should have a meaningful impact on pathology. The logical synthesis of these prior findings, the established role of the *Postn*^*+*^ cell target in TAC and the validated function of Cd248 in ischemia, created a strong expectation of efficacy for our experiment.

The absence of a fibrosis phenotype following Cd248 deletion in *Postn*^*+*^ cells suggests that the functions of fibroblast Cd248 in the pressure-overload model may be not directly associated with ECM remodeling. We rigorously excluded technical limitations as explanations for the null phenotype. First, regarding “target burden,” we integrated our scRNA-seq data with published MI datasets and confirmed that the abundance of *Cd248*^*+*^ fibroblasts in our TAC model (∼35%) is comparable to that in ischemic models (∼15–30%), ruling out insufficient target density. Second, regarding genetic validity, we utilized the same Postn-Cre strategy that successfully rescued the MI phenotype in previous studies [14] and we confirmed that our driver efficiently ablated CD248 protein in *Postn*^*+*^ myofibroblasts. Finally, we validated our in vitro findings using a second, independent siRNA set to rule out off-target effects. Collectively, these data support the biological validity of our findings.

To understand why the robust phenotypic changes observed in vitro failed to translate into in vivo antifibrotic efficacy, we performed comprehensive transcriptomic profiling. This analysis revealed a critical “molecular uncoupling” in the function of CD248. While Cd248 knockdown significantly downregulated the “actomyosin” module, suppressing genes essential for contraction and migration like *Myl9*, *Cnn1*, and *Wnt11*, it failed to suppress the “matrix” module, leaving the expression of bulk collagen genes (*Col1a1, Col3a1*) and *Postn* intact. This indicates that CD248 functions primarily as a regulator of cytoskeletal dynamics rather than a master switch for ECM synthesis. Consequently, in the pressure-overloaded heart, Cd248-deficient fibroblasts likely retain their full capacity to secrete collagen, driving extensive interstitial fibrosis even if their migratory or contractile properties are impaired.

Single-cell transcriptomic reanalysis revealed that Cd248 is expressed across multiple populations; notably CD248 expression in *Postn*^*+*^ fibroblast in the TAC model are enriched for chemotaxis and migration, not ECM organization programs. These data indicate that Cd248 has clear cell-autonomous effects in mesenchymal cells in vitro but that deleting Cd248 solely in *Postn*^*+*^ myofibroblasts is insufficient to blunt pressure-overload fibrosis in vivo. Moreover, our single-cell analysis confirms that Cd248 is also persistently expressed in *Pdgfrβ*^+^ mural cells and proliferating endothelial cells, indicating that pericytes and endothelial cells are also a constitutive source of Cd248 in the TAC heart. Future studies employing pericyte-specific lineage-tracing or endothelial-specific tracing and gene deletion are required to fully elucidate the role of CD248 in pressure-overload induced cardiac injury.

The null phenotype reported here stands in notable contrast to these positive findings and is therefore highly informative. This discrepancy suggests that the function of Cd248 on *Postn*^*+*^ fibroblasts is profoundly context-dependent, differing between ischemic injury and chronic pressure overload. While Cd248 appears crucial for the pathological activity of fibroblasts in post-infarction remodeling, our data demonstrate it is dispensable within the same cell lineage when the injury stimulus is sustained hemodynamic stress. This highlights the distinct biological mechanisms underlying different fibrotic diseases and cautions against the assumption that anti-fibrotic targets are universally applicable. The divergence in efficacy in MI and TAC models likely stems from the distinct pathophysiology of the models. Mechanistically, Li et al. identified that CD248 promotes fibrosis primarily by stabilizing TGFβRI and upregulating ACKR3 to facilitate T-cell retention and immune-fibroblast crosstalk. Our single-cell analysis similarly indicated that *Cd248*^*+*^ fibroblasts in the TAC model were enriched for chemotaxis signatures rather than direct ECM production programs. However, unlike the MI model where inflammation is paramount, the recruitment of immune cells in our chronic TAC model was sparse and unchanged by Cd248 deletion. This suggests that the CD248-dependent immune-modulatory axis, while critical for ischemic repair, is redundant or non-essential in the context of pressure-overload induced compensated hypertrophy. Furthermore, we observed a regulatory disconnect where Cd248 is robustly upregulated by TAC in vivo but downregulated by TGFβ1 in vitro. This suggests that Cd248 is not a direct downstream effector of the canonical TGFβ fibrotic axis but is likely driven by other microenvironmental cues (e.g., hypoxia or mechanical stress) that are distinct in pressure overload. Finally, it is important to contextualize our findings within the specific physiology of the C57BL/6 J mouse strain used in this study. Consistent with the well-documented resistance of C57BL/6 J mice to the transition from hypertrophy to heart failure, our TAC cohort exhibited a phenotype of compensated hypertrophy. Notably, the EF in our 8-week TAC model remained normal, and we did not observe an abnormal EF even up to 16 weeks. While we cannot rule out a role for CD248 in decompensated failure in other strains (e.g., C57BL/6 N), our data unequivocally show that it is not required for the establishment of hypertrophy or the extensive interstitial fibrosis that characterizes this compensated phase. These results indicate that while CD248 has clear cell-autonomous effects in mesenchymal cells in vitro, deleting Cd248 solely in *Postn*^*+*^ myofibroblasts is insufficient to blunt pressure-overload fibrosis in vivo.

From a translational perspective, targeting CD248 with neutralizing antibodies has shown considerable promise as an anti-fibrotic strategy in a range of preclinical settings. Efficacy has been demonstrated not only in animal models of ischemic heart injury [13], [14] but also in fibrotic conditions of the kidney [19], liver [20], and skin [21]. This body of evidence, combined with the favorable safety profile of the CD248-targeting antibody Ontuxizumab in oncology trials [10], has generated significant interest in its potential application for non-malignant fibrotic diseases. However, our findings introduce a critical note of caution. The lack of efficacy in the pressure-overload model, despite the positive results in other pathologies, underscores that our understanding of CD248's function is incomplete. Therefore, a better and more in-depth investigation of the context-dependent role of CD248 in various pathological conditions is critical to successfully guide and refine the clinical translation of these promising anti-fibrotic therapies.

### 4.1 Limitations of the study

Our findings are constrained by a single-sex design (male mice), and lineage restriction to *Postn*^*+*^ myofibroblasts, which spares mural cells and endothelial cells indicated by our scRNA-seq reanalysis. Future work using pericyte- or endothelial-restricted deletions, female cohorts, will better define cell-type and sex-specific roles of CD248 in pressure-overload remodeling. For single-cell RNAseq data analysis, we must interpret these quantitative readouts with caution. It is well-established that the sample preparation for single-cell RNA sequencing can introduce significant selection bias, specifically, the enzymatic dissociation required to isolate cells is markedly less efficient in dense, collagen-rich fibrotic tissue compared to normal myocardium. This differential recovery can lead to the underrepresentation of matrix-embedded myofibroblasts in injury samples. Therefore, direct quantitative comparisons of cell proportions between Sham and TAC/MI samples, or across different injury models, may be confounded by technical artifacts and might not accurately reflect the true in situ biological burden.

## 5 Conclusion

In summary, our findings indicate that the specific ablation of CD248 in the Postn^+^ myofibroblast lineage does not substantially mitigate fibrosis or cardiac dysfunction in this murine model of pressure-overload cardiomyopathy. These results highlight the context-dependent function of CD248, suggesting its role is not universally conserved across different etiologies of cardiac injury. Therefore, the future development of anti-fibrotic therapies targeting this molecule should consider the specific disease model and may need to address its function in other cell types, such as pericytes, to achieve therapeutic efficacy.

## Materials availability

This study did not generate new unique reagents.

## CRediT authorship contribution statement

**Donghua Li:** Methodology, Investigation, Formal analysis, Data curation. **Dongmei Zhong:** Methodology, Investigation, Formal analysis, Data curation. **Tian Tan:** Methodology, Investigation, Formal analysis, Data curation. **Zhilei Huang:** Methodology, Investigation, Data curation. **Mingyue Wu:** Methodology, Investigation. **Yongshan Liu:** Methodology, Investigation. **Yalin Zhang:** Methodology, Investigation. **Chen Liu:** Resources. **Jie Xu:** Writing – review & editing, Writing – original draft, Project administration, Funding acquisition, Conceptualization. **Fu-Li Xiang:** Writing – review & editing, Writing – original draft, Project administration, Conceptualization.

## Declaration of Generative AI and AI-assisted technologies in the writing process

During the preparation of this manuscript the authors used Google Germini 2.5-pro for word editing only to improve readability and language. After using this tool/service, the authors reviewed and edited the content as needed and takes full responsibility for the content of the published article.

## Declaration of competing interest

None.

## Data Availability

Public datasets analyzed include GSE166403 (mouse TAC scRNA-seq) CRA022616 (mouse MI scRNA-seq), CRA005739 (mouse MI scRNA-seq), and a published human HCM snRNA-seq dataset (https://figshare.com/collections/single-nucleus_RNA-seq_and_spatial_transcriptomics_of_hypertrophic_cardiomyopathy/5777948/2). All source data underlying figures and custom analysis scripts will be deposited to Zenodo at acceptance and linked in the final article. RNAseq data of cultured mCMC generated in this study will be provided per request to Fu-li Xiang.

## Appendix A Supplementary data

Supplementary figures Image 1

Supplementary material Image 2
