# Supplementary material for: Deletion of Cd248 in Postn+ myofibroblast fails to attenuate pressure-overload induced cardiac remodeling and fibrosis in mice
Source: J Mol Cell Cardiol Plus. 2026 Mar 4;15:100837. doi: 10.1016/j.jmccpl.2026.100837 (PMC12994045; doi:10.1016/j.jmccpl.2026.100837)

**Supplemental Figure 1.** Single-cell analysis reveals enriched CD248 expression in pericytes and fibroblasts from human hypertrophic cardiomyopathy hearts.

(A, B) UMAP visualization of cardiac cells from healthy control and hypertrophic cardiomyopathy (HCM) human hearts, color-coded by cell type (A) or sample group (B). (C) UMAP feature plot showing normalized CD248 expression across all cells. (D) Bar plot quantifying the percentage of CD248⁺ cells within each cell lineage.

**Supplemental Figure 2.** Cd248 mRNA expression is upregulated in murine models of cardiac injury.

(A) Relative mRNA expression of Cd248 across various organs from healthy adult C57BL/6J mice, demonstrating the highest basal expression in the heart, lung, and kidney. (B) Myocardial Cd248 mRNA expression is significantly increased 1 week after pressure overload induced by transverse aortic constriction (TAC) compared to sham-operated controls. (C) Myocardial Cd248 mRNA expression is significantly increased in the infarct border zone 3 days after myocardial infarction (MI) compared to sham-operated controls. Data are presented as mean ± SEM; n=3–6 mice per group. **P* < 0.05 vs. sham, determined by an unpaired Student’s *t-test*.

**Supplemental Figure 3.** Validation of the purity of primary cultured mCFs.

(A) Representative immunofluorescence images of cultured primary adult mCFs stained for the fibroblast marker Vimentin (green, bottom row) and the endothelial marker CD31 (green, top row). Nuclei are counterstained with DAPI (blue). The images demonstrate that 100% of the cultured cells are positive for Vimentin, while very few CD31-positive endothelial cells are detected, confirming high fibroblast purity. Scale bars, 50 µm. (B) Transcriptional profiling of marker gene expression in cultured mCFs by bulk RNA-sequencing (FPKM values). The data confirm robust expression of fibroblast-lineage markers (e.g., *Vim, Dcn, Col1a1, Col1a2, Col3a1*) and minimal or negligible expression of markers for other cardiac cell types, including immune cells (*Ptprc, Ctss, Lyz2, Ms4a7, Fcgr3, C1qc*), endothelial cells (*Pecam1, Kdr, Cdh5, Egfl7*), smooth muscle cells/pericytes (*Tagln, Myh11, Des, Cspg4, Rgs5, Kcnj8, Abcc9*), and cardiomyocytes (*Tnni3, Tnnt2, Tnnc1*).

**Supplemental Figure 4.** Cd248 knockdown efficacy and its effect on extracellular matrix gene expression *in vitro*.

(A) Quantitative RT-PCR analysis confirming the efficient knockdown of Cd248 mRNA in primary mCFs. Cells were transfected with scrambled siRNA (si-Scrambled, blue bars) or Cd248-targeting siRNA (si-Cd248, red bars) and cultured with or without TGF-β1 stimulation. Cd248 expression is significantly reduced in the knockdown group under both baseline and stimulated conditions. Note that TGF-β1 treatment itself downregulates Cd248 expression in control cells. (B-D) Quantitative RT-PCR analysis of key fibrosis-related genes: *Col3a1* (B), *Col1a1* (C), and *Postn* (D). While Cd248 knockdown significantly reduces *Col3a1* expression (B), it does not significantly alter the expression of *Col1a1* (C) or *Postn* (D) in response to TGF-β1 stimulation. (E) Representative immunofluorescence images validating the knockdown of CD248 protein. Cells were stained for CD248 (green) and Periostin (Postn, red), with nuclei counterstained with Hoechst (blue). The images confirm that transfection with si-Cd248 effectively abolishes CD248 protein expression (bottom row) compared to the scrambled control (top row) in TGF-β1-stimulated cells, while Periostin protein expression remains robust and unchanged. Data are presented as mean ± SEM; n=6 independent experiments. Two-way ANOVA followed by Tukey’s multiple comparisons test was used. * P < 0.05 vs. si-Scrambled Control; # P < 0.05 vs. si-Scrambled + TGF-β1. Scale bar, 50 µm.

**Supplemental Figure 5.** Validation of Cd248 function using a second, independent set of siRNAs.

(A) Sequences of the second set of independent siRNAs (si-Cd248 set 2) targeting murine Cd248 used to rule out off-target effects. (B) Representative immunofluorescence images of primary mCFs transfected with scrambled control or the second set of Cd248-targeting siRNAs. Cells were stimulated with vehicle or TGF-β1 (5 ng/mL) for 24 hours and stained for α-SMA (green) and nuclei (blue). Consistent with the first siRNA set, transfection with si-Cd248 set 2 markedly reduced the formation of α-SMA stress fibers in response to TGF-β1. Scale bar, 50 µm. (C) Quantification of myofibroblast activation and gene expression. Data are presented as mean ± SEM. Two-way ANOVA followed by Tukey’s multiple comparisons test. * P < 0.05 vs. Control; # P < 0.05 vs. si-Scrambled + TGF-β1.

**Supplemental Figure 6.** Characterization of the TAC model and CD248 upregulation in C57BL6J mice.

(A) Validation of the TAC model using pulsed-wave Doppler echocardiography. Representative images show a dramatic increase in peak aortic flow velocity from ~1000 mm/s in sham-operated mice to >4400 mm/s two weeks after TAC, confirming significant pressure overload. (B) Representative Picro-Sirius Red staining of left ventricular sections showing robust interstitial and perivascular fibrosis (red) in WT hearts 6 weeks post-TAC compared to Sham controls. (C) Long-term echocardiographic follow-up of WT mice subjected to TAC. While left ventricular posterior wall (LVPW) and anterior wall (LVAW) thickness significantly increased by 16 weeks, indicating sustained hypertrophy, the Ejection Fraction (EF) and Fractional Shortening (FS) remained preserved (>50%) at both 1 week and 16 weeks post-TAC. This confirms the model represents a state of compensated hypertrophy rather than decompensated heart failure. * P < 0.05 vs Sham. (D) Representative immunofluorescence images of WT heart sections at baseline and 6 weeks post-TAC. Staining for CD248 (red) and the endothelial marker CD31 (white) reveal a marked upregulation of CD248 protein in the interstitium of the remodeling heart compared to the low basal expression in healthy tissue. Scale bar, 50 µm.

**Supplemental Figure 7.** Specificity of the Postn-Cre driver and validation of *in vivo* cell-type specific deletion.

(A) Evaluation of Cre recombinase specificity using the Postn-MCM; LSL-tdTomato reporter line. Representative immunofluorescence images of heart sections 2 and 6 weeks after TAC show that RFP (tdTomato) expression is restricted to the interstitium and does not overlap with the cardiomyocyte marker Cardiac Troponin T (CTNT), confirming the absence of "leaky" recombination in cardiomyocytes. (B) Validation of efficient CD248 deletion in myofibroblasts. Immunofluorescence staining 8 weeks post-TAC demonstrates that while CD248 co-localizes with Periostin (Postn) in the fibrotic areas of control mice (PostnMCM^+/-^), it is successfully ablated in the Postn^+^ cells of the knockout mice (PostnMCM^+/-^; Cd248^fl/fl^). (C) assessment of vascular CD248 expression. Co-staining for CD248 and the endothelial marker CD31 reveals that CD248 expression persists in CD31^+^ vascular structures in the knockout hearts, confirming that the deletion is strictly limited to the Postn^+^ fibroblast lineage and spares the endothelial compartment. Scale bars as indicated.

**Supplemental Figure 8.** Post-operative survival and aortic peak velocity in *Postn*^+^ myofibroblast-specific Cd248 deletion mice.

(A) Kaplan-Meier survival analysis over an 8-week period following TAC surgery. There was no significant difference in survival between the control (blue line; n=11) and Cd248-cKO mice (red line; n=8), as determined by the log-rank test. (B) Quantification of aortic peak velocity by Doppler echocardiography 2 weeks after TAC surgery. The data shows a comparable degree of stenosis and pressure overload in both Cd248-cKO mice and their littermate controls. Each dot represents an individual animal; bars indicate mean ± SEM, n=7-11.

**Supplemental Figure 9.** *Postn*^+^ myofibroblast-specific deletion of Cd248 does not alter myocardial deformation in response to pressure overload.

Speckle-tracking echocardiography was performed at baseline and 8 weeks after TAC. Control littermates (blue bars) are compared with Cd248-cKO (red bars). (A, B) Quantification of global radial strain rate (A) and reverse global radial strain rate (B). (C, D) Quantification of global longitudinal strain (C) and reverse global longitudinal strain (D). No significant differences in any strain parameter were observed between genotypes at either baseline or 8 weeks post-TAC. Data are presented as mean ± SEM; n=5-7, each dot represents an individual animal. Two-way ANOVA was performed, followed by Tukey’s multiple comparison post-hoc test. **P* < 0.05 vs. baseline.

**Supplemental Figure 10.** Divergent regulation of chemotactic markers by CD248 in pressure overload.

(A) Heatmap showing the relative expression of chemotaxis- and signaling-associated genes (*Ackr3, Dpp4, S100a4, Nbl1, Sema3c, Ackr2, Fgf18, Pgf*) in four fibroblast subpopulations from Sham and TAC hearts. The *Postn^+^Cd248^+^* population (in both Sham and TAC) exhibits higher expression of these markers compared to the *Postn^+^Cd248^-^* subset. (B) Quantitative RT-PCR analysis of these signature genes in cultured mCFs transfected with Cd248 siRNA or scrambled control, with or without TGF-β1 stimulation. Unlike the in vivo association, Cd248 knockdown in vitro failed to downregulate these genes (e.g., *Pgf, Sema3c, S100a4, Nbl1*) and even slightly increased *Ackr3* expression, indicating that CD248 does not directly regulate this chemotactic program downstream of TGF-β1 in this context. Data are mean ± SEM. Two-way ANOVA was performed, followed by Tukey’s multiple comparison post-hoc test. # P < 0.05 vs. si-Scrambled + TGF-β1.

**Supplemental Figure 11**. Assessment of inflammatory cell infiltration in the compensated hypertrophic heart.

(A) Representative immunofluorescence images of left ventricular heart sections stained for the pan-leukocyte marker CD45 (red) and nuclei (Hoechst, blue) in PostnMCM^+/-^ (control) and PostnMCM^+/-^;Cd248^fl/fl^ (knockout) mice 8 weeks post-TAC. Very few CD45^+^ leukocytes were detected in either genotype. (B) Representative immunofluorescence images of heart sections stained for the macrophage marker CD68 (red) and the endothelial marker GSL-I B4 (green). Macrophage infiltration remains sparse and comparable between groups. (C) Positive control immunofluorescence staining of Spleen and Liver sections, demonstrating robust detection of CD68 to validate antibody specificity. Scale bars as indicated.

**Supplemental Figure 12.** Integrated single-cell analysis comparing CD248 abundance in sham groups of TAC versus MI models.

(A) Integrated UMAP visualization of cardiac interstitial cells combining a TAC dataset (GSE166403, blue) with two independent Myocardial Infarction (MI) datasets (CRA022616, red; CRA005739, green). The panels display the integrated clustering by cell type (left) and sample origin (right). (B) Quantification of the percentage of cells positive for Cd248 (left), Cd248/Postn double-positive (middle), and Postn (right) across cell lineages. (C) Violin plots of unique molecular identifier (UMI) counts (nCount_RNA) for each dataset, confirming comparable technical quality and sequencing depth across the integrated samples.

**Supplemental Figure 13**. Integrated single-cell quantification of CD248 and Periostin expression burden in TAC versus MI models.

Bar graphs quantifying the percentage of cells positive for Cd248 (left panel), co-expressing Cd248 and Postn (middle panel), and positive for Postn (right panel) across identified cardiac cell types. The analysis integrates single-cell RNA-sequencing data from the current TAC study (GSE166403; Sham of TAC [red], TAC 14D [green]) and a published MI study (CRA005739; Sham of MI [blue], MI 14D [purple]).

**Supplemental Figure 14**. Cd248 knockdown fails to reverse established myofibroblast activation in vitro.

(A) Experimental design: Primary mCFs were pre-treated with TGF-β1 (5 ng/mL) for 8 hours to initiate myofibroblast differentiation prior to transfection with control (si-Scrambled) or Cd248-targeting siRNA (si-Cd248). (B) Representative images and quantification of the percentage of cells containing organized α-SMA stress fibers. Myofibroblast activation is indicated by the formation of α-SMA stress fibers (green); nuclei are counterstained with Hoechst (blue). Scale bar, 50 µm. (C) Relative mRNA expression of *Acta2* (α-SMA) and *Cd248*. Data are presented as mean ± SEM. Two-way ANOVA followed by Tukey’s multiple comparisons post-hoc test. * P < 0.05 vs. Control. # P < 0.05 vs. si-Scrambled + TGF-β1.


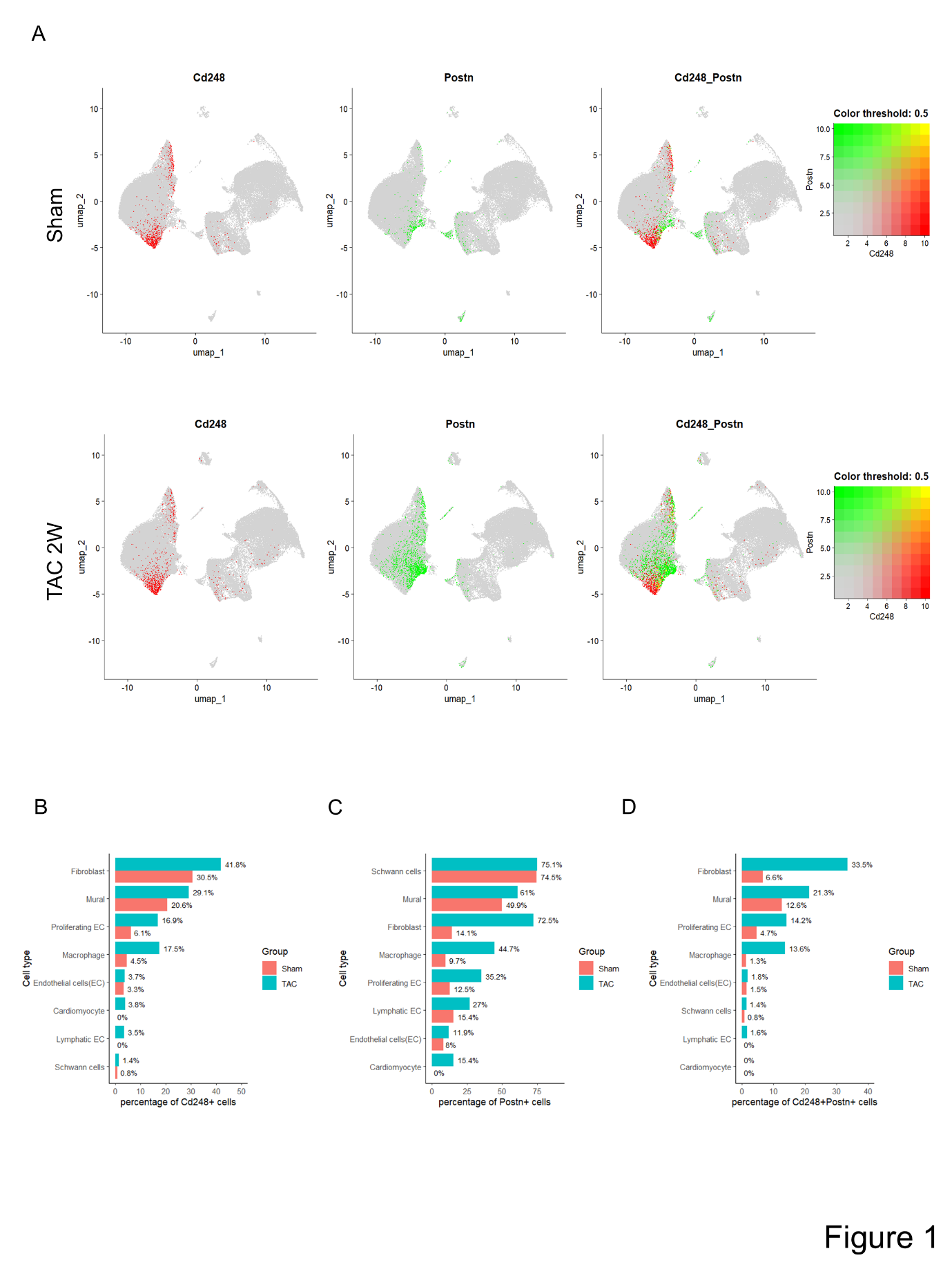


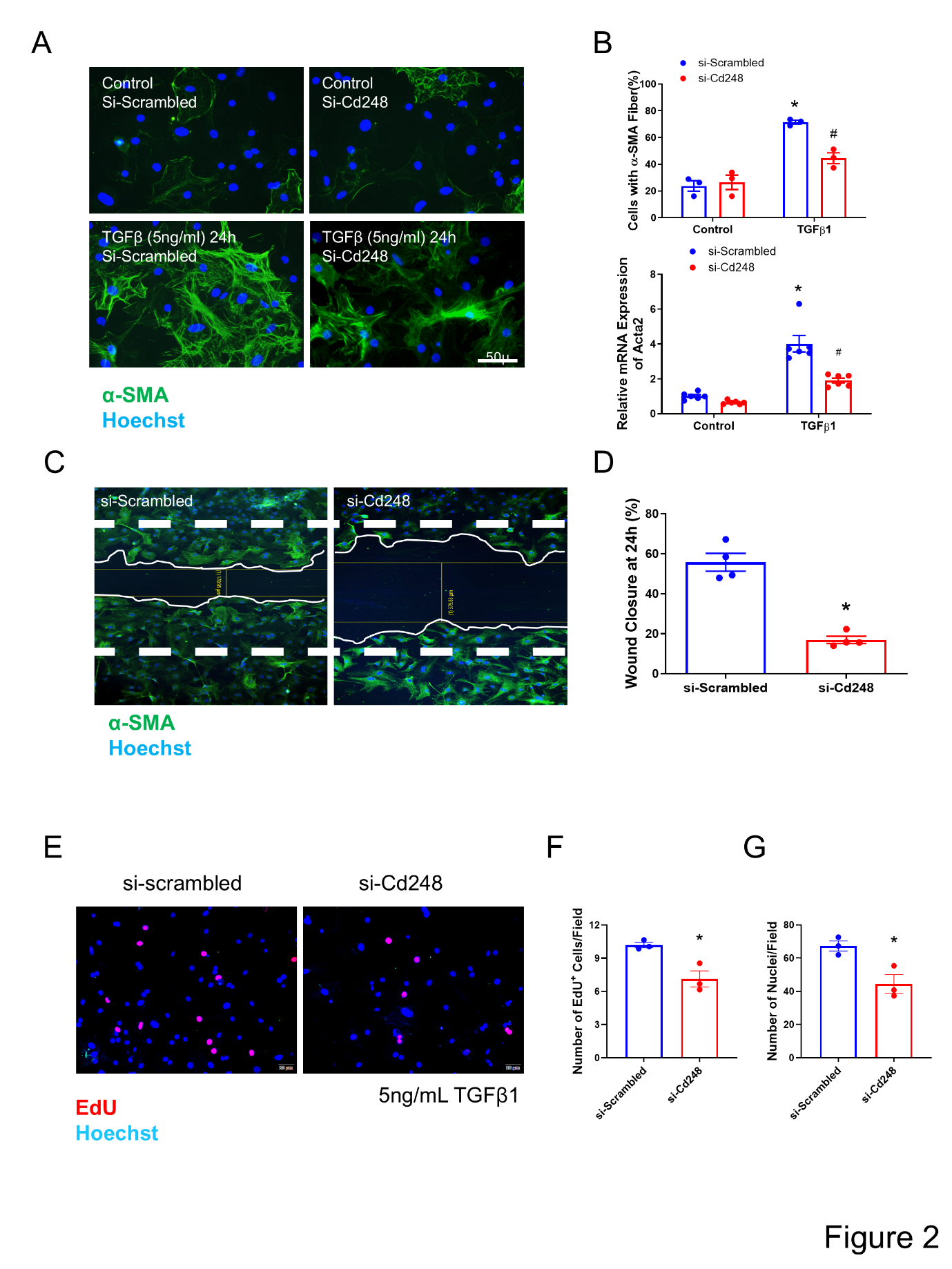


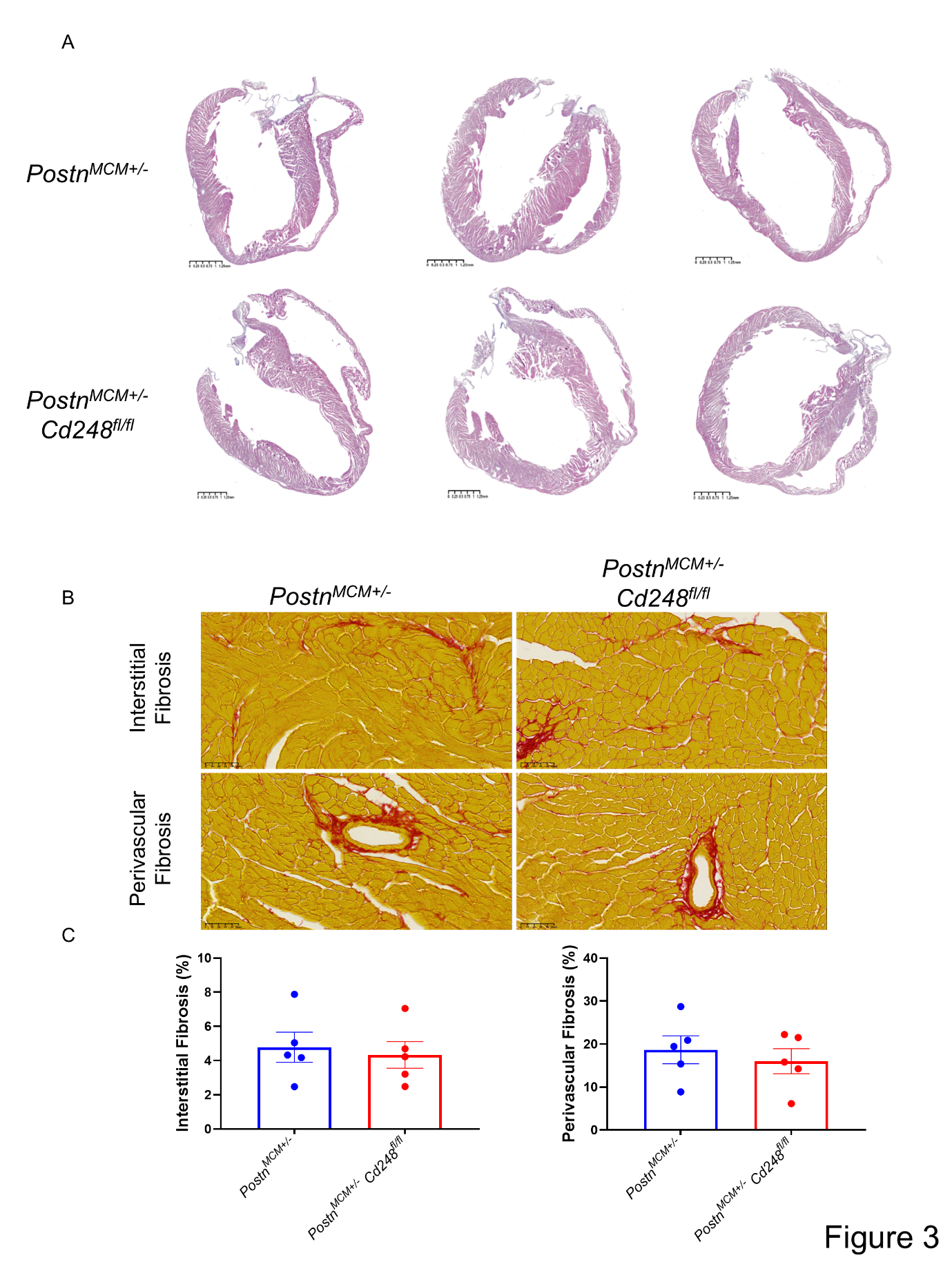


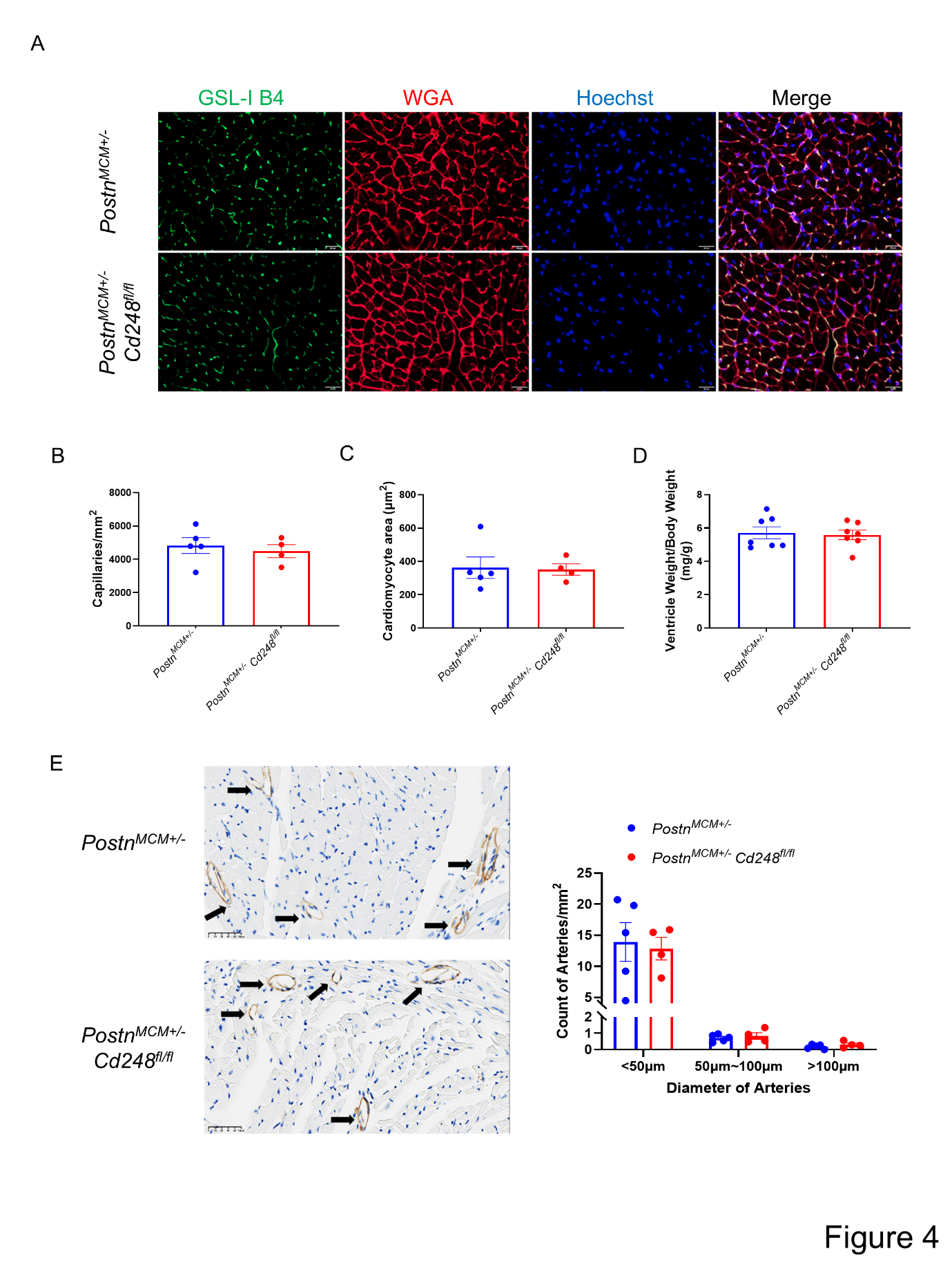


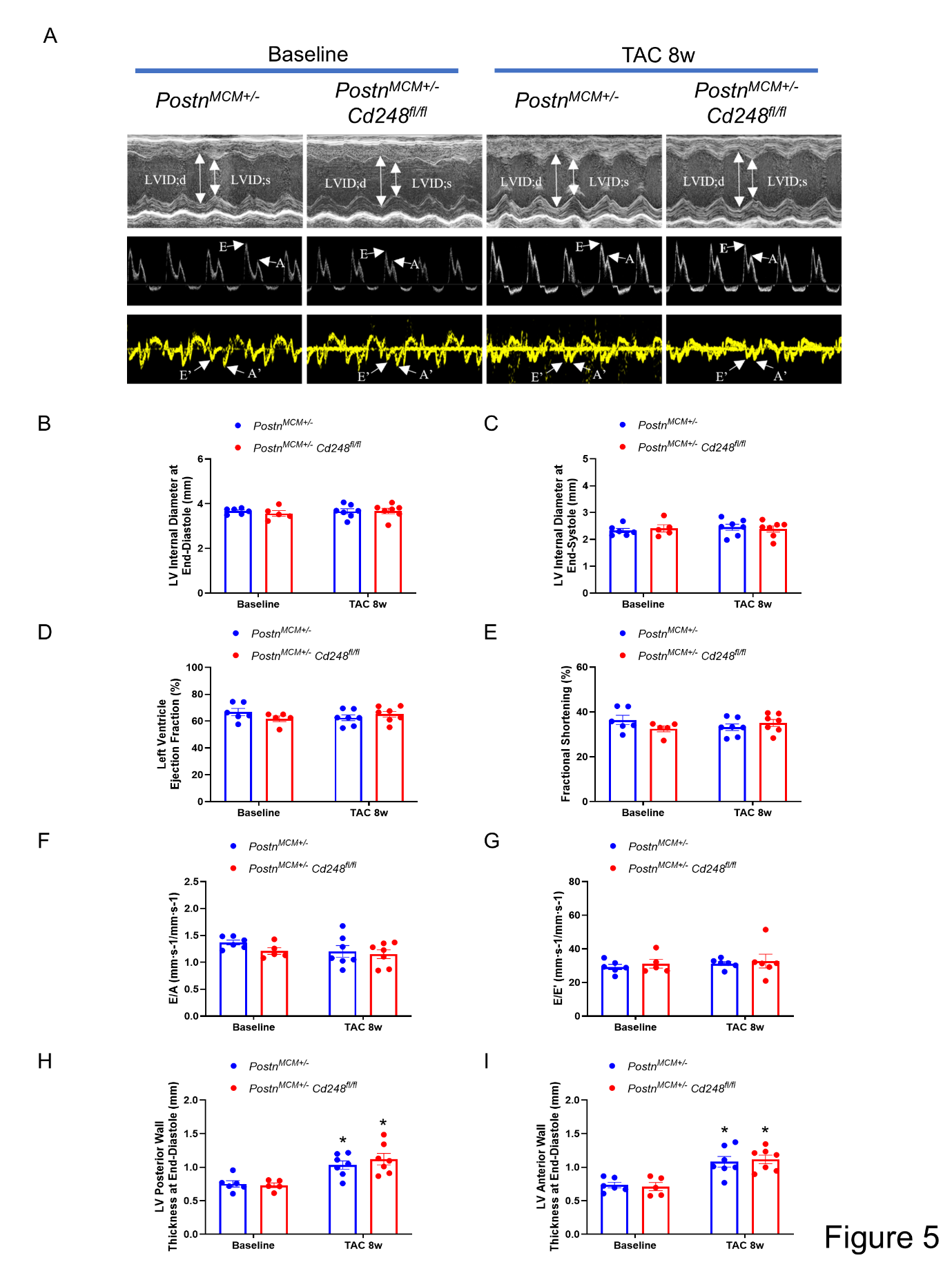


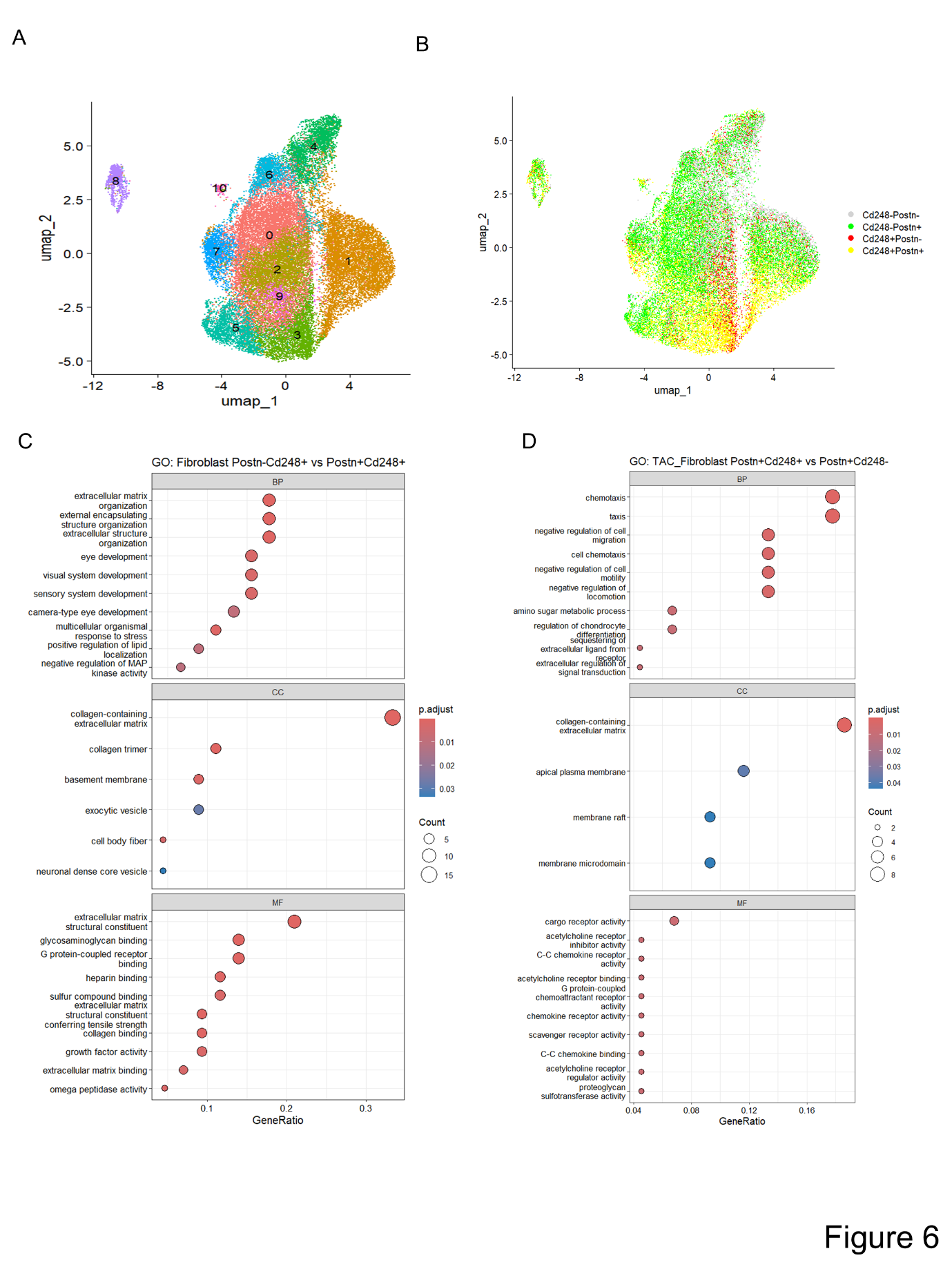


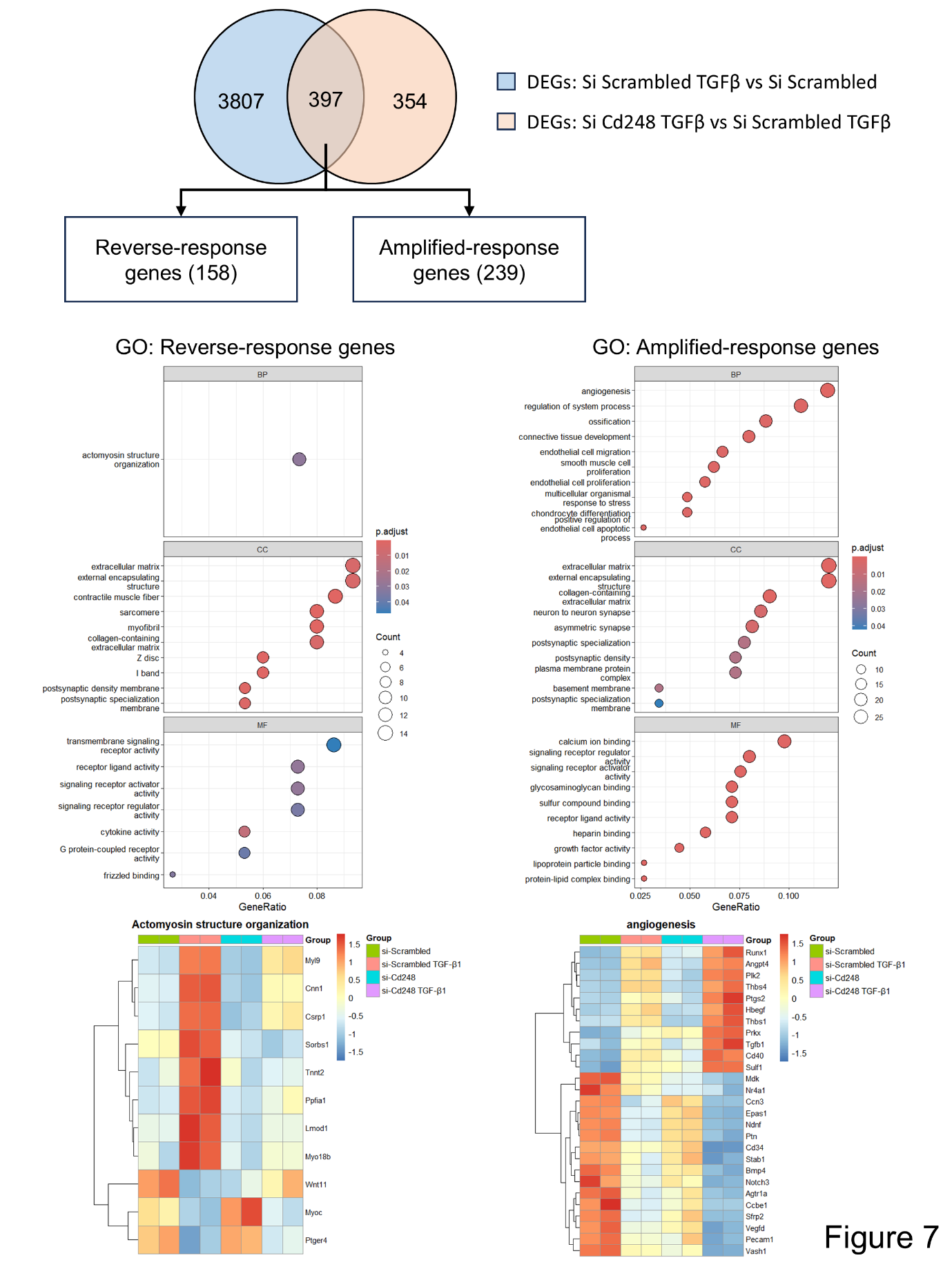


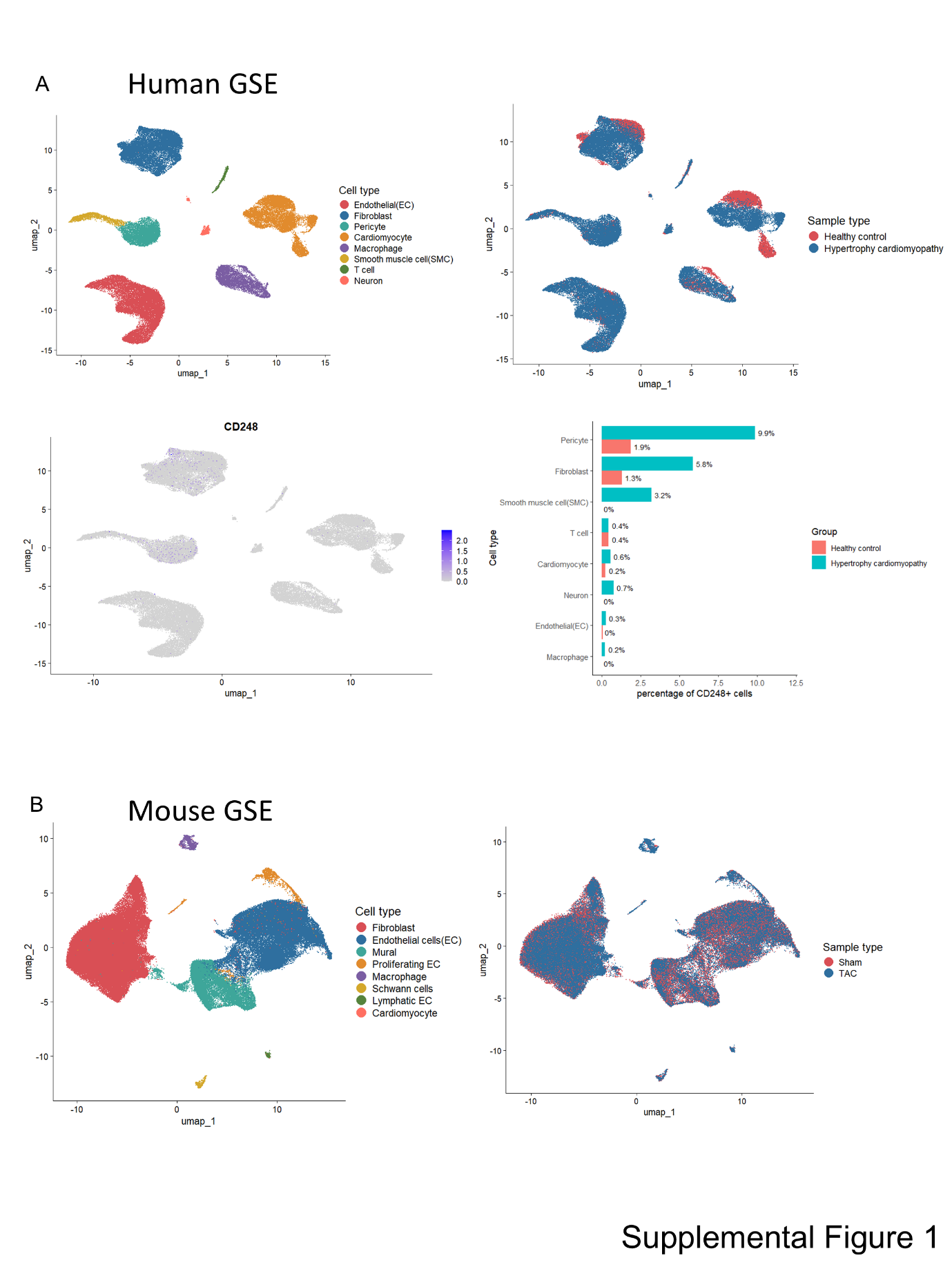


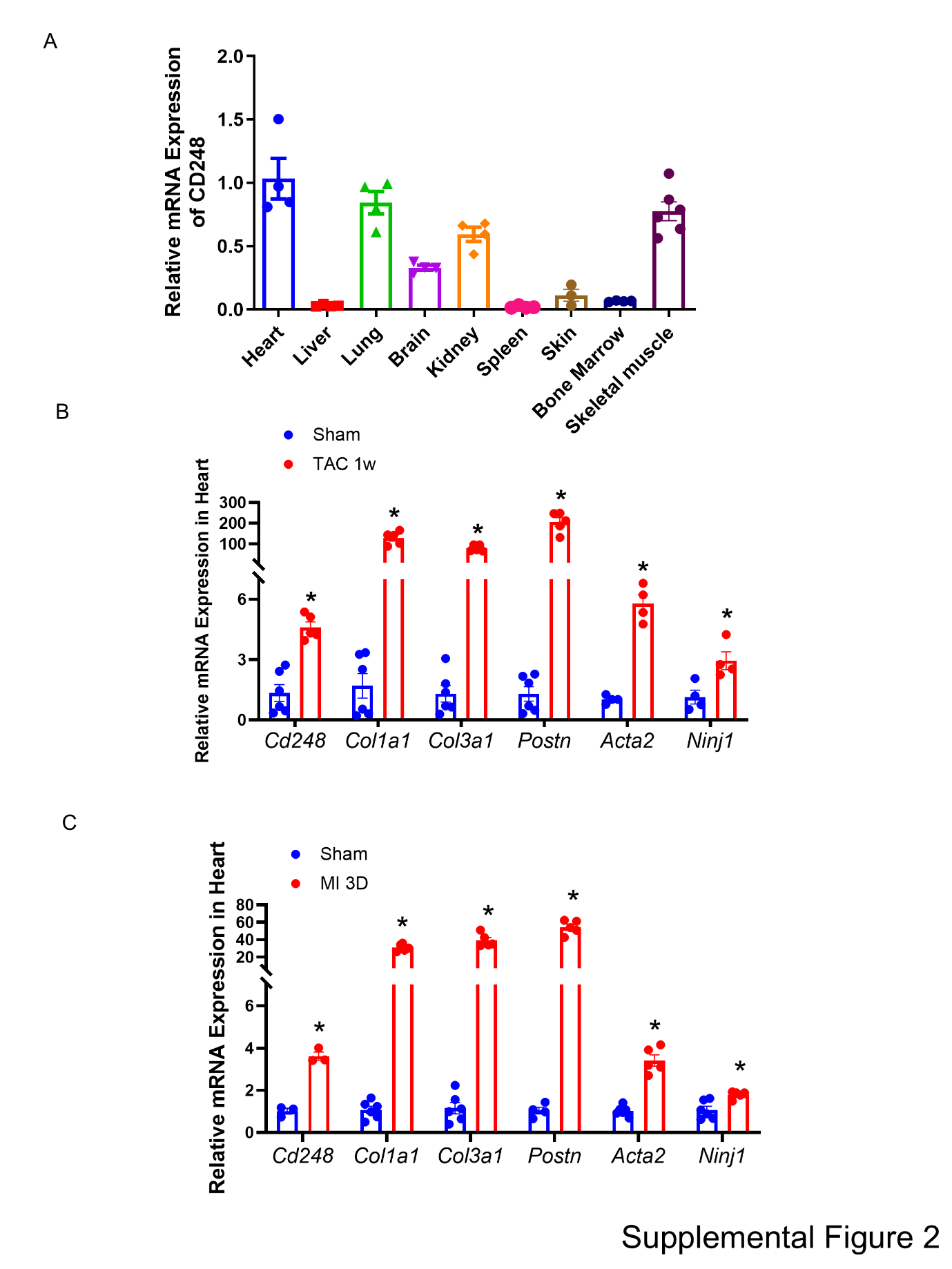


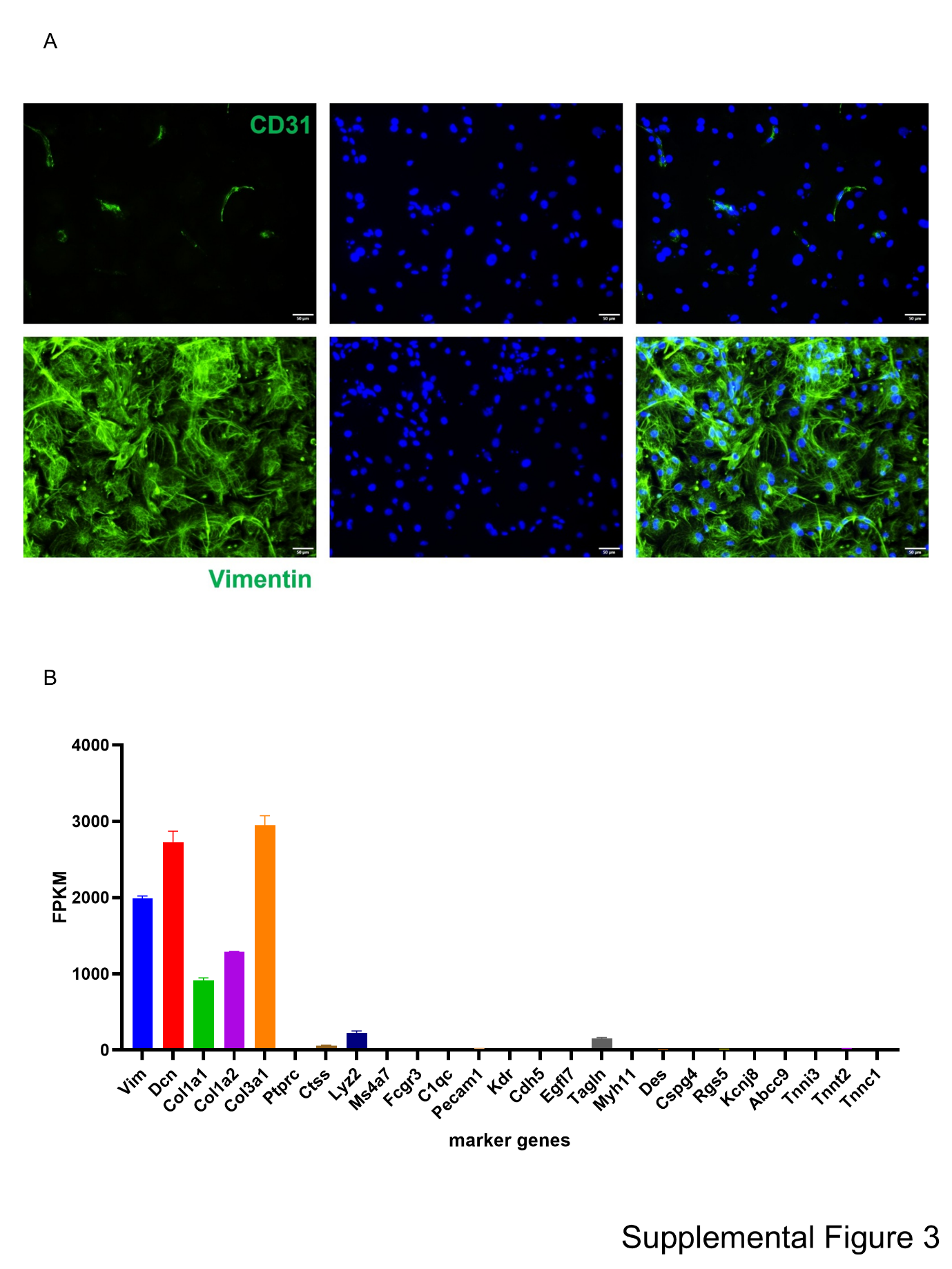


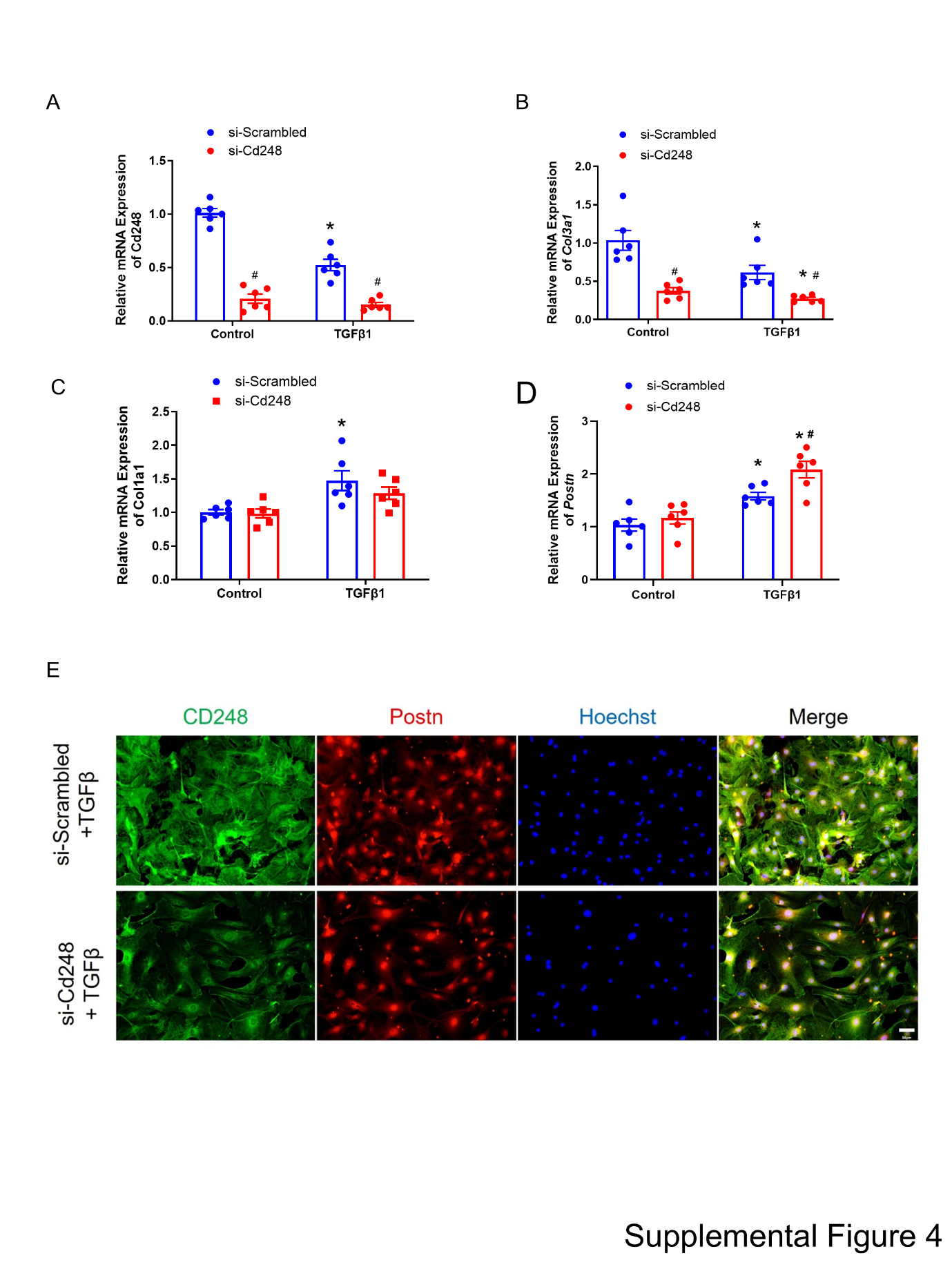


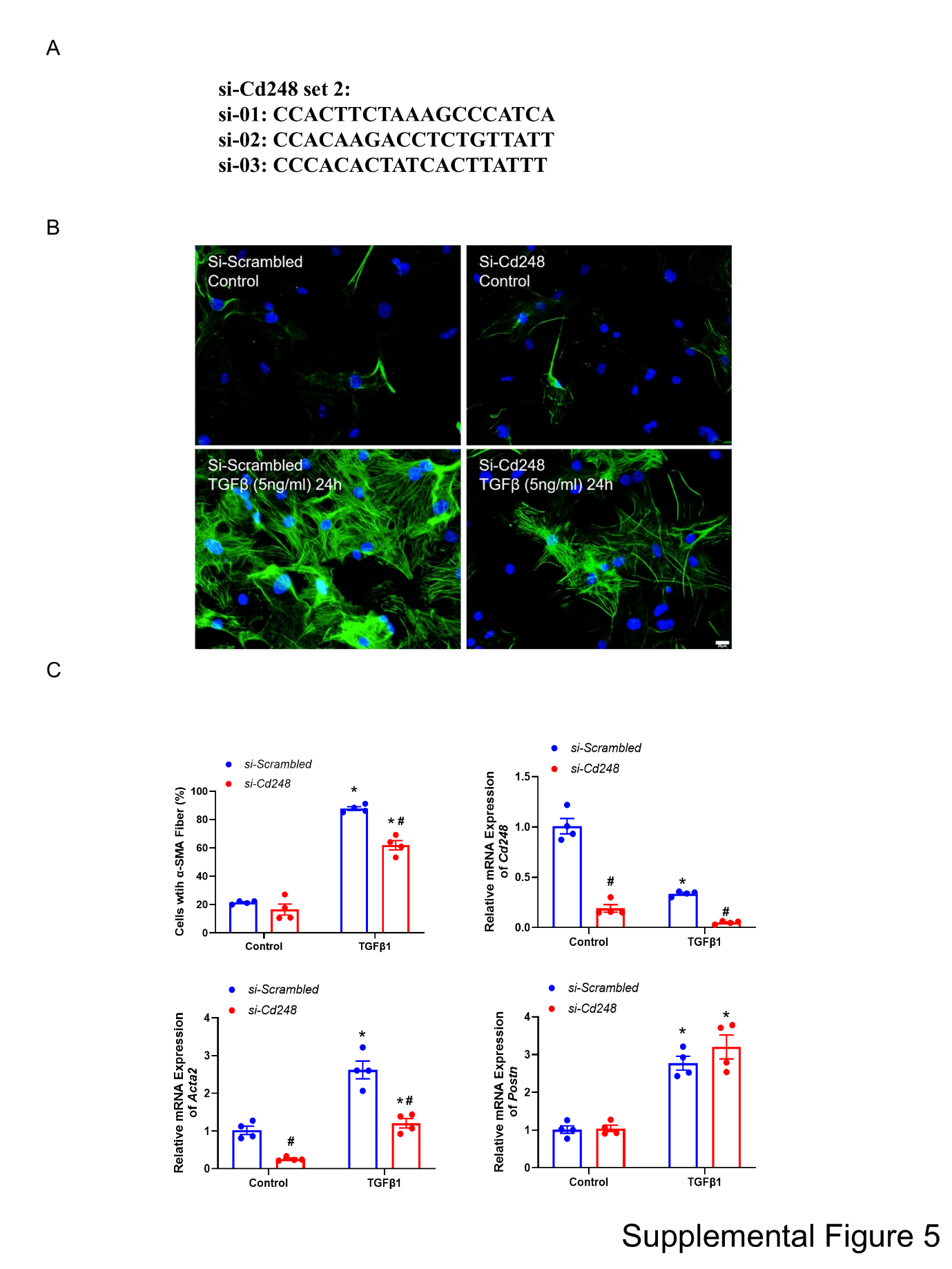


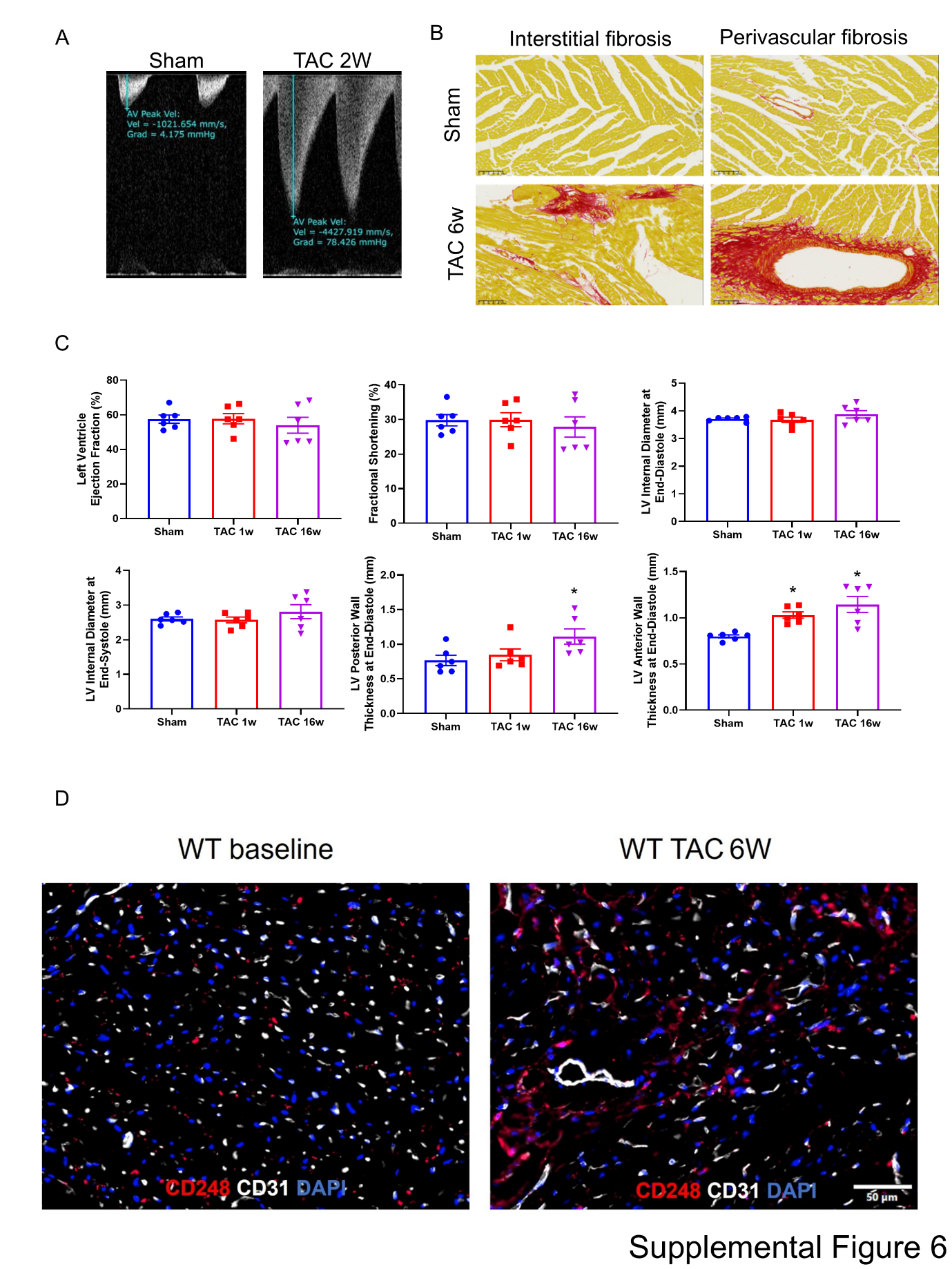


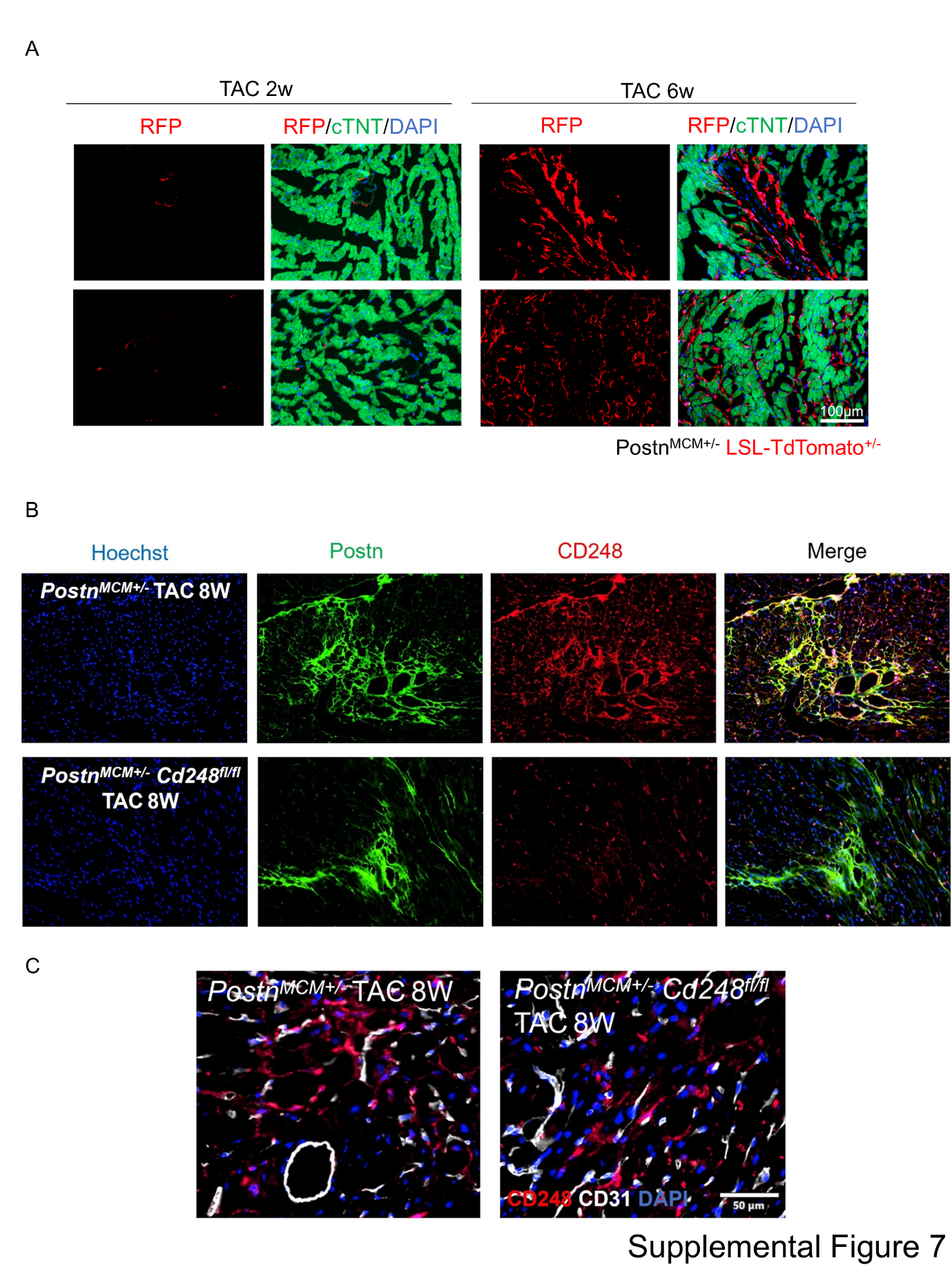


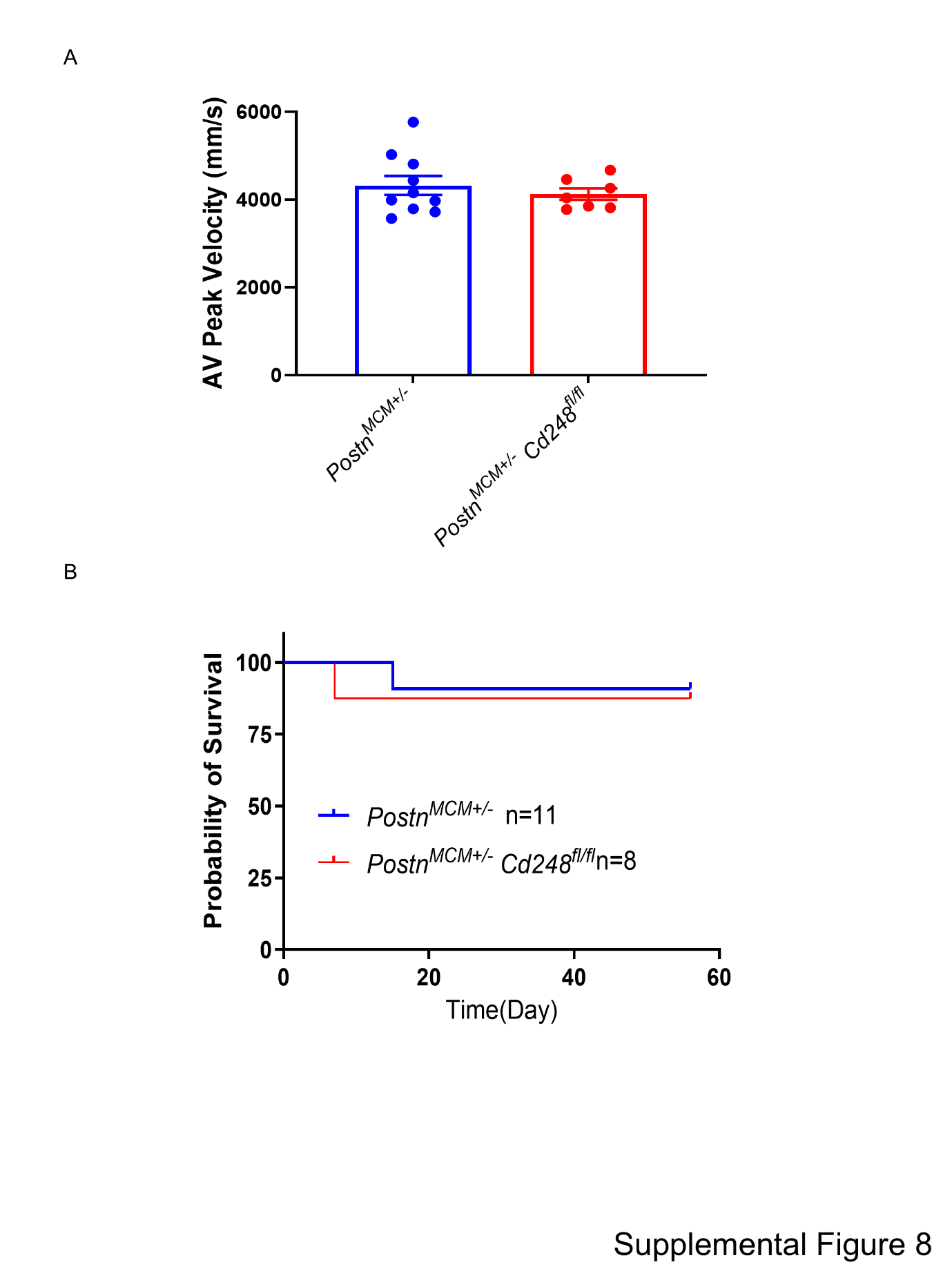


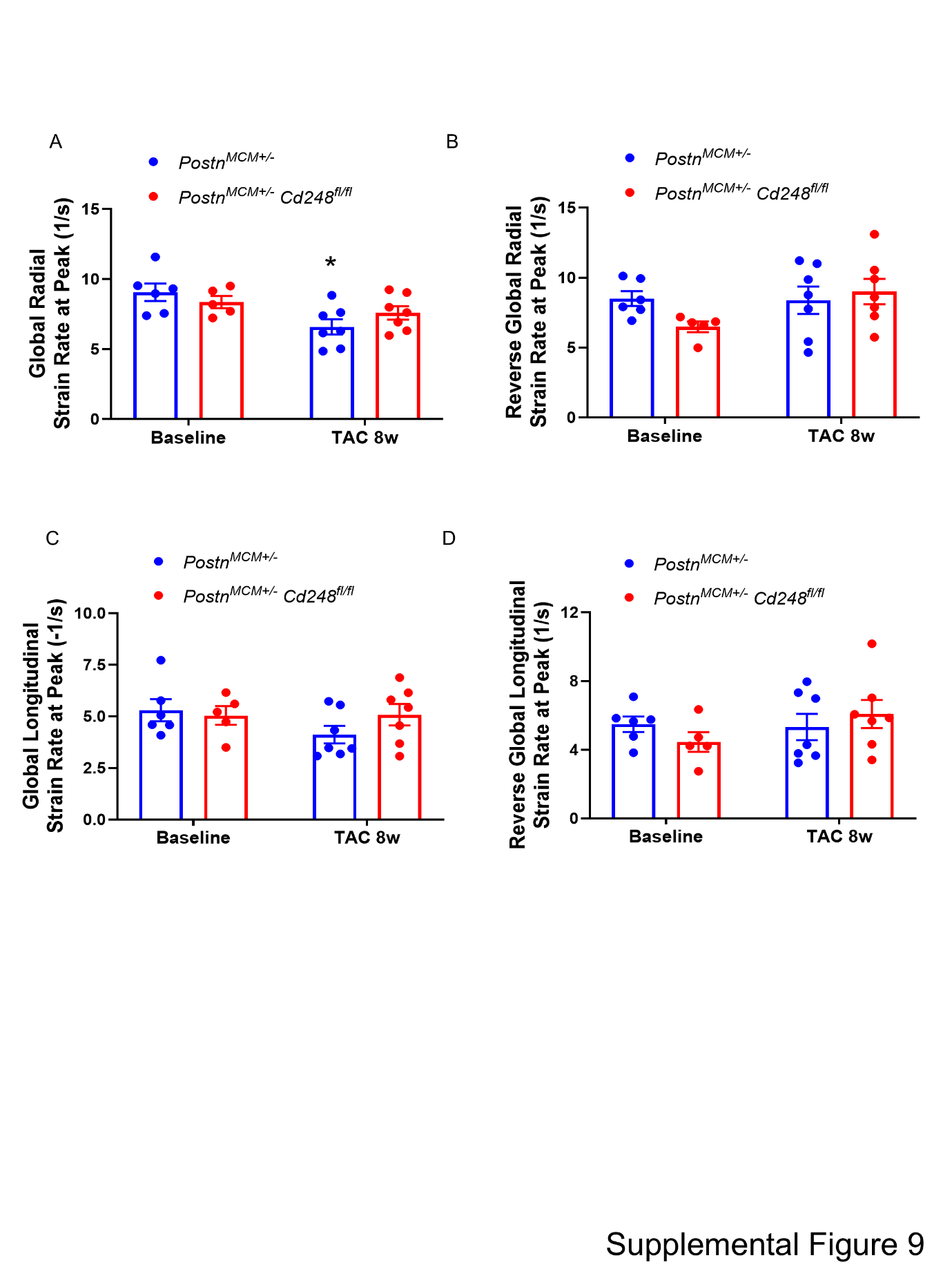


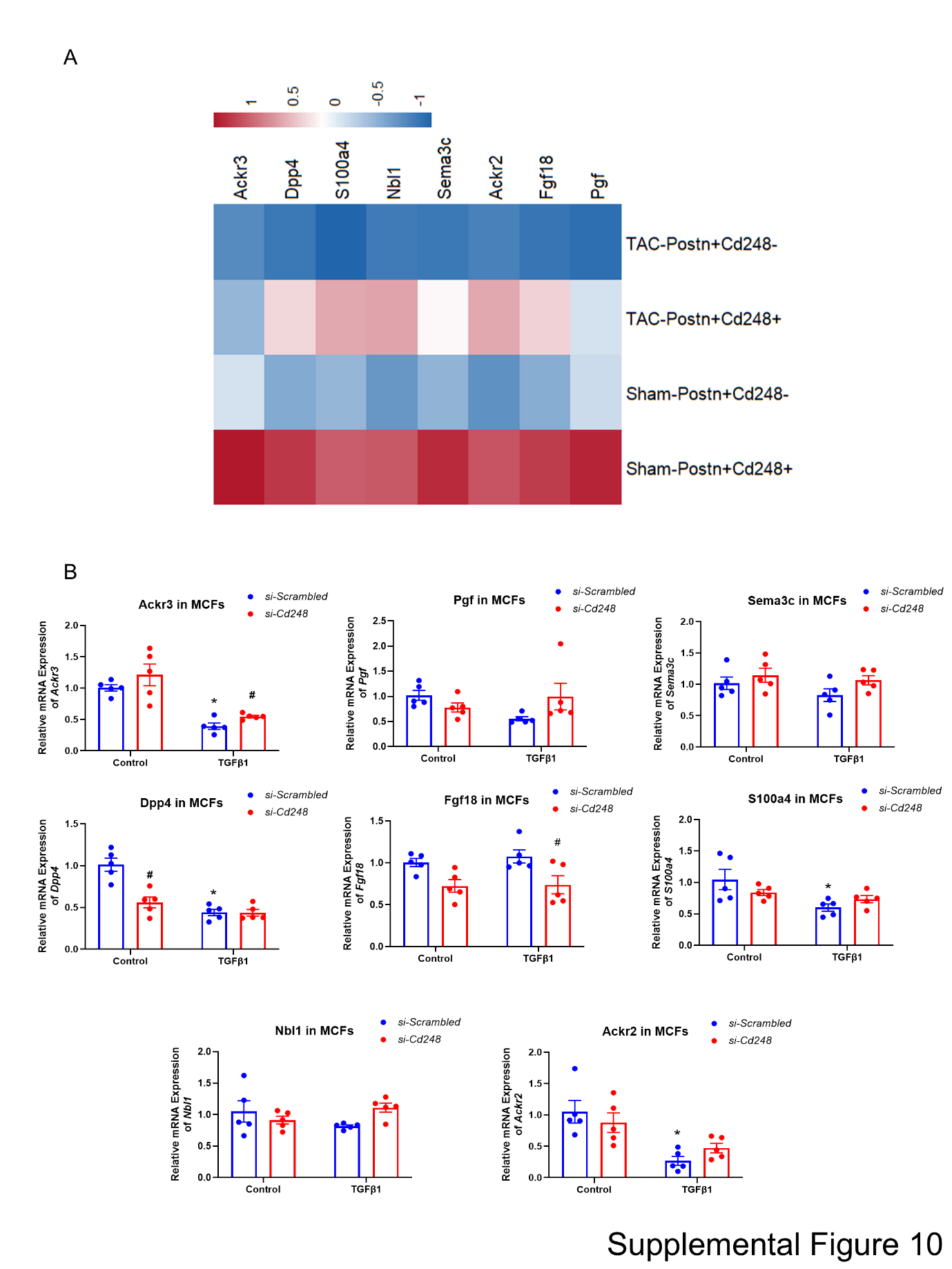


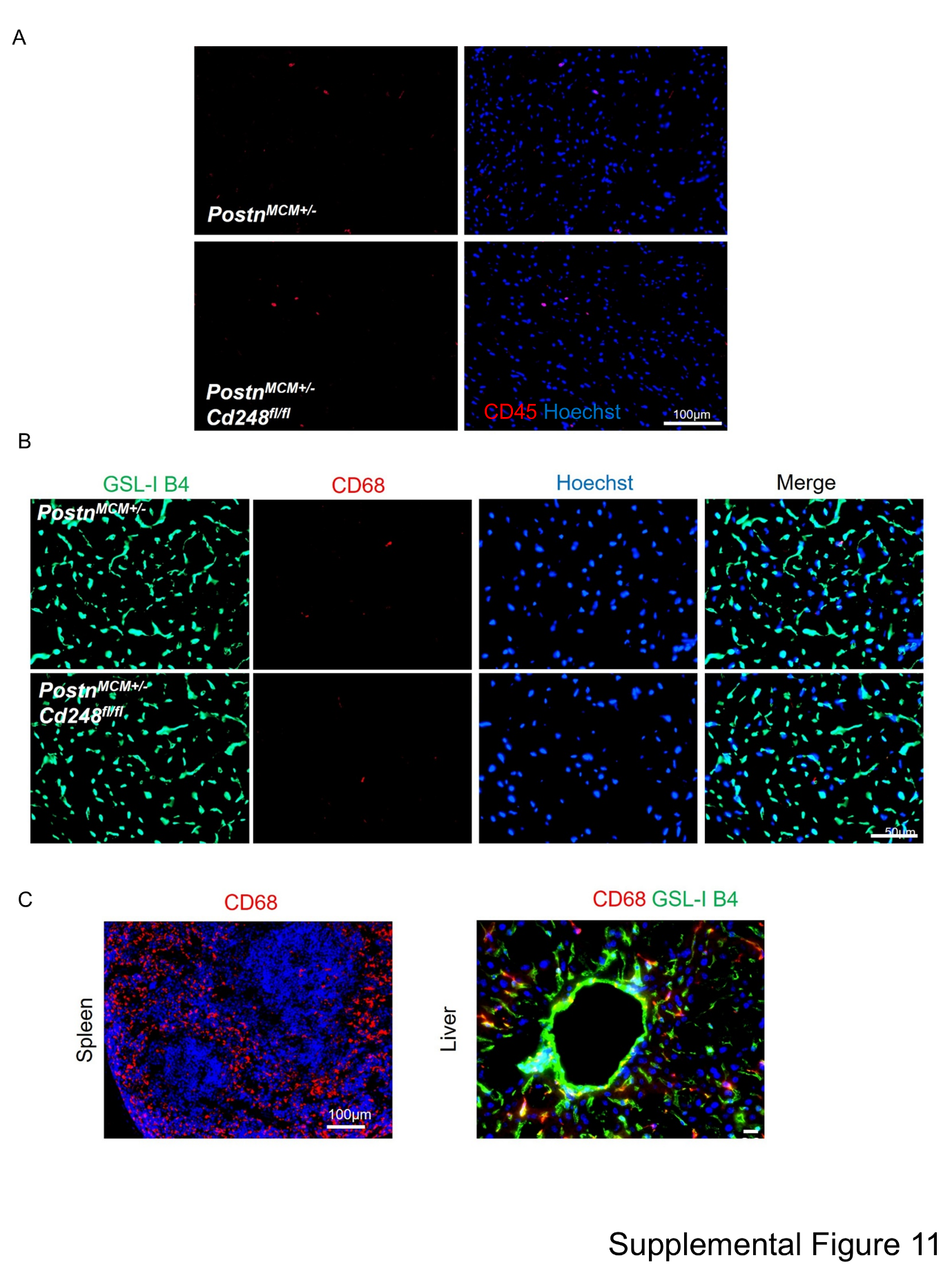


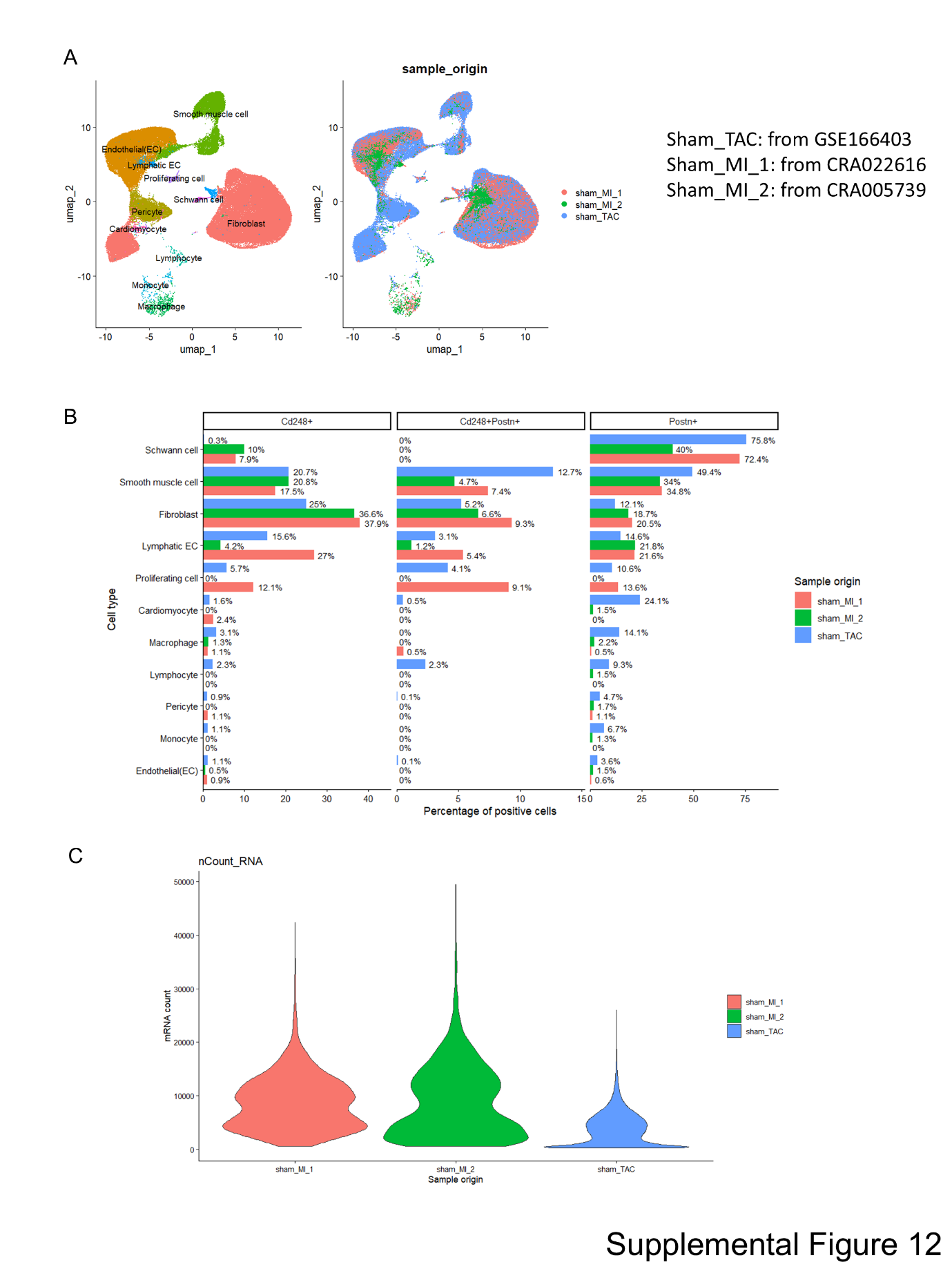


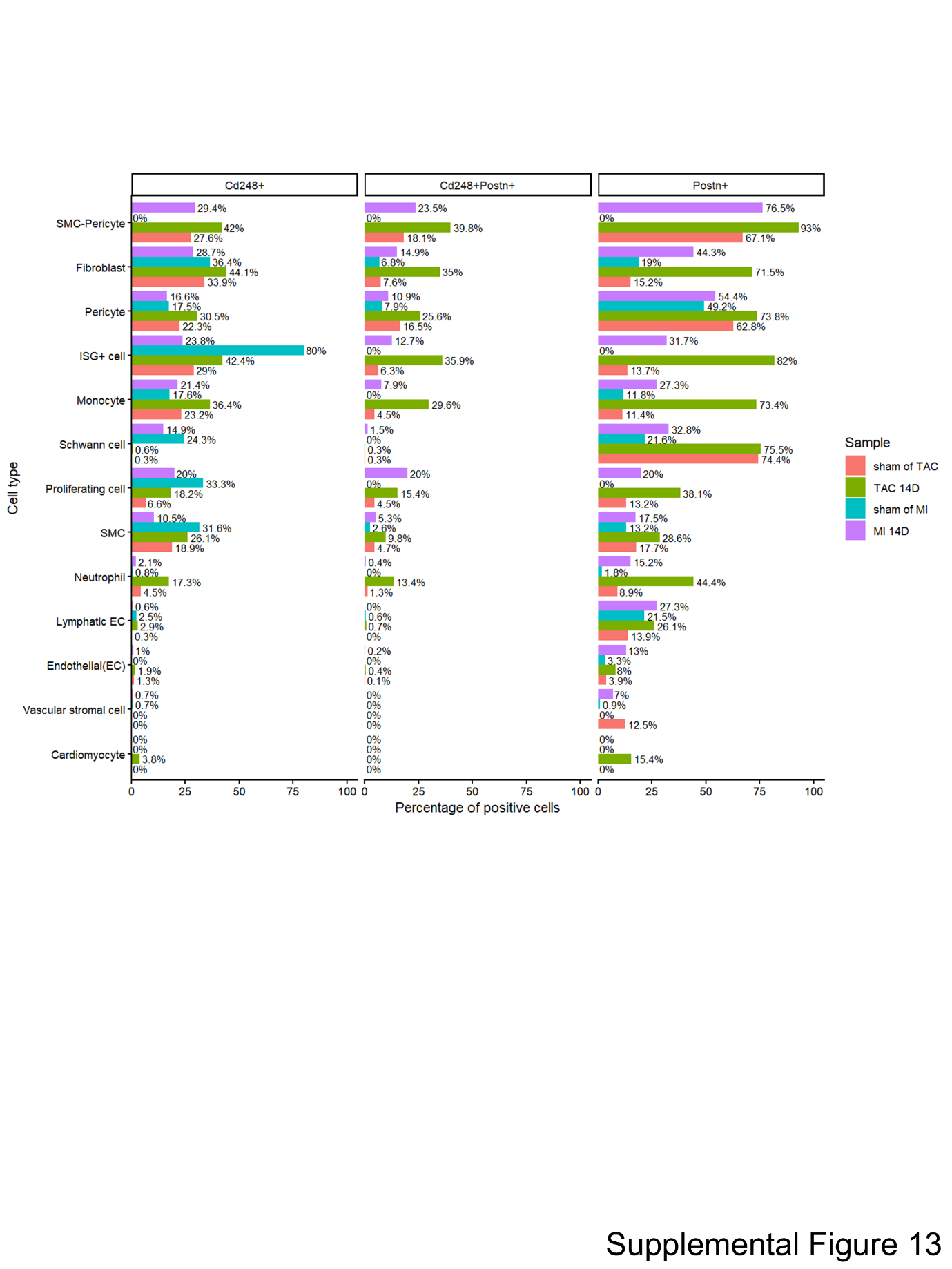


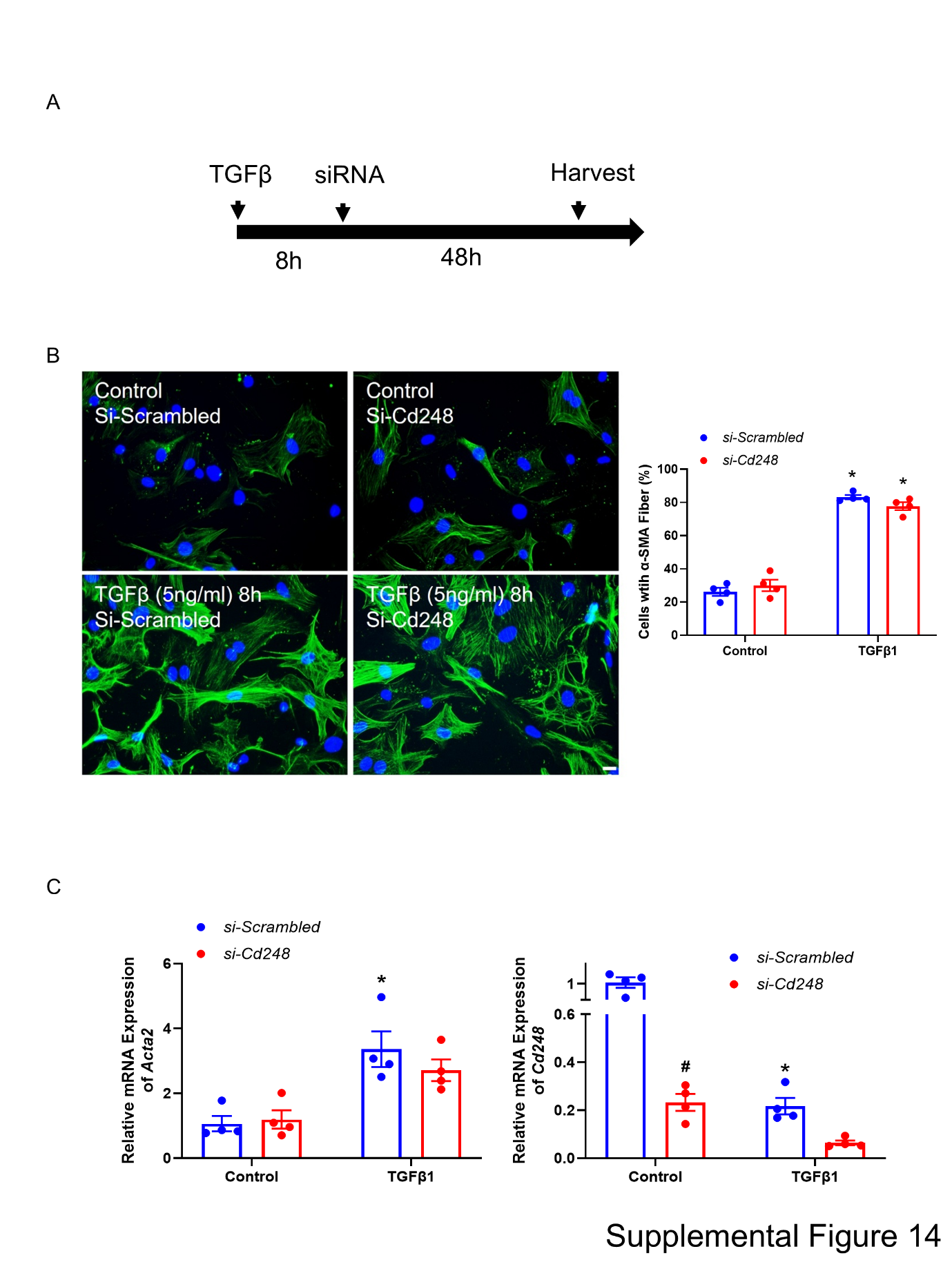

Supplement: Supplementary file 1 — Supplementary figures [file mmc1.docx]
