# Supplementary material for: Deletion of Cd248 in Postn+ myofibroblast fails to attenuate pressure-overload induced cardiac remodeling and fibrosis in mice
Source: J Mol Cell Cardiol Plus. 2026 Mar 4;15:100837. doi: 10.1016/j.jmccpl.2026.100837 (PMC12994045; doi:10.1016/j.jmccpl.2026.100837)
Supplement: Supplementary file 2 — Supplementary material [file mmc2.docx]

**Animals and Husbandry**

All animal experiments were approved by the Sun Yat-sen University Animal Care and Use Committee (SYSU-IACUC-2024-002343). Myofibroblast-specific inducible *Cd248* knockout mice (*PostnMCM+/-;Cd248fl/fl*) and their corresponding controls (*PostnMCM+/-*, The Jackson Laboratory, 029645 RRID:IMSR_JAX:029645)^[1, 2]^ were generated on a C57BL/6J background (RRID:IMSR JAX:000664). To validate Cre recombinase activity, *PostnMCM+/-* mice were crossed with a TdTomato reporter line containing a Loxp-Stop-Loxp cassette. All mice were housed in a specific pathogen-free (SPF) facility on a 12-hour light/dark cycle with ad libitum access to food and water. All surgical and experimental procedures were performed on male mice aged 10-14 weeks.

Animal Welfare, Refinement Measures, Adverse Events, and Humane Endpoints

All animal procedures were approved by the Sun Yat-sen University Animal Care and Use Committee (SYSU-IACUC-2024-002343) and complied with institutional and national guidelines. Predefined refinement measures to minimize pain, suffering, and distress, adverse‑event reporting, and humane endpoints were in place before study initiation.

(a) Interventions to reduce pain, suffering, and distress (Refinement)

Anesthesia & peri‑operative care: TAC and sham surgeries were performed under inhalational anesthesia (isoflurane in oxygen) at a surgical plane verified by pedal‑withdrawal and palpebral reflexes. Body temperature was maintained with a thermostatic warming surface throughout anesthesia and recovery; ophthalmic lubricant was applied to prevent corneal desiccation. Aseptic technique, microsurgical instruments, and atraumatic tissue handling were used. Incisions were closed in layers with absorbable sutures.

Recovery & housing: Mice recovered in warmed cages under direct observation until ambulatory, with easy access to hydration gel and softened chow on the cage floor. Animals were singly housed for up to 24–72 h after surgery if needed to prevent interference with the incision, then returned to group housing with enrichment as soon as compatible with welfare.

Procedural minimization: Non‑survival procedures were conducted under deep anesthesia. Noninvasive assessments (e.g., echocardiography) were performed under light, brief inhalational anesthesia with active warming to reduce physiologic stress.

Personnel & monitoring: Only trained personnel performed TAC. Intra‑ and postoperative checks included respiratory rate/effort, mucous‑membrane color, activity, posture, grooming, incision integrity, hydration status, and body weight.

(b) Humane endpoints, monitoring criteria, and frequency

Humane endpoints were prespecified in the IACUC protocol. Any animal meeting euthanasia criteria (eg. ≥20% body weight loss) was promptly euthanized using CO₂ inhalation followed by a secondary physical method (e.g., bilateral thoracotomy or cervical dislocation) in accordance with institutional guidance.

Monitoring criteria and frequency: Immediate post‑op (0–4 h): Continuous observation until fully ambulatory and thermally stable. Early post‑op (Days 0–3): 2–3 checks/day for pain, respiration, hydration, incision, and weight (weighed at least daily). Subacute phase (Days 4–14): Daily welfare checks; weight at least 3×/week. Chronic phase (> Day 14 to endpoint): ≥3×/week checks; escalate to daily if any clinical concern arises. After any procedure (e.g., echocardiography): Additional same‑day welfare check

**Experimental Design: Gender Selection, Sample size, Randomization, and Blinding**

All surgical and experimental procedures were performed exclusively on male mice aged 10-14 weeks to reduce biological variability potentially caused by hormonal cycles and to limit the total number of animals required to achieve statistical significance. For comparisons between genotypes, littermate controls were used whenever possible. We selected a small sample size because the phenotype associated with the CD248 KO mice was evaluated in TAC in vivo model for the first time in the present study, and therefore, the initial intention was to gather basic evidence regarding the cardiac phenotypes. Within each genotype, mice were randomly allocated to either the transverse aortic constriction (TAC) or sham surgery group. Key outcome assessments, including the analysis of echocardiographic data and the quantification of histological fibrosis and cardiomyocyte size, were performed by investigators who were blinded to the genotype and surgical group of the animals

**Transverse Aortic Constriction (TAC) Surgery**

Pressure overload was induced via TAC surgery^[1]^. Mice were anesthetized with 2% isoflurane, intubated with a 24-gauge catheter, and mechanically ventilated (130 breaths/min, ~200-250 μL tidal volume, Kent Scientific, RoVent® Jr.). Body temperature was maintained at 37°C. A left thoracotomy was performed at the second intercostal space to expose the aortic arch. A 6-0 silk suture was passed under the aorta between the brachiocephalic and left common carotid arteries. A 27-gauge blunt needle was placed alongside the aorta, and the suture was tied snugly around both the needle and the aorta. The needle was then promptly withdrawn to create a defined stenosis. The chest and skin were closed in layers. Sham-operated mice underwent the same procedure without aortic ligation. A pre‑specified inclusion threshold (the peak blood flow velocity across the aortic arch >3.5 m/s) was used to exclude unsuccessful TAC surgery. For inducible gene deletion, all mice in the *PostnMCM* cohorts were fed a diet containing tamoxifen (400 mg/kg, TAM, Xietong Shengwu, SSY11221) starting on the day of surgery and continuing until the experimental endpoint.

**Myocardial Infarction (MI) Surgery**

To induce myocardial infarction^[3]^, mice are anesthetized with 2% isoflurane, intubated, and mechanically ventilated. Body temperature is maintained at 37°C throughout the procedure. A left thoracotomy is performed at the fourth intercostal space to expose the heart. The pericardium is gently opened to visualize the left anterior descending (LAD) coronary artery. An 8-0 silk suture is passed under the LAD, approximately 2-3 mm from its origin. The artery is then permanently ligated by tightening the suture. Successful occlusion is confirmed by the immediate and sustained blanching of the anterior ventricular wall distal to the ligature. The chest and skin are then closed in layers. Sham-operated animals undergo the same procedure without the final ligation of the LAD artery.

**Echocardiography**

Transthoracic echocardiography was performed on anesthetized (about 2% isoflurane) mice before surgery and at endpoints using a Vevo 3100 high-resolution imaging system (VisualSonics)^[1]^. A Mx550D transducer was used for functional and dimensional analysis. Left ventricular (LV) dimensions, wall thickness, fractional shortening (FS), and ejection fraction (LVEF) were derived from M-mode images obtained in the parasternal short-axis view. Diastolic function, including early (E) and late (A) mitral inflow velocities, was assessed using pulsed-wave Doppler from the apical four-chamber view. Myocardial strain was analyzed from B-mode cine loops using speckle-tracking analysis. To confirm the severity of the constriction, a Mx250D transducer was used to measure the peak blood flow velocity across the aortic arch via pulsed-wave Doppler one day post-surgery. All measurements were averaged from at least three consecutive cardiac cycles.

For a more sensitive assessment of cardiac muscle performance, strain analysis was performed using speckle-tracking software (Vevo Vasc, VisualSonics)^[4]^. High-frame-rate B-mode cine loops were acquired from the parasternal long-axis view. The software tracks the motion of natural acoustic speckles within the myocardium frame-by-frame throughout the cardiac cycle to quantify regional and global deformation. Key systolic parameters, including global longitudinal strain (GLS) and global radial strain (GRS), were calculated to assess myocardial shortening and thickening, respectively. Strain rates were also determined to evaluate the speed of myocardial deformation. All strain measurements were averaged from at least three consecutive cardiac cycles.

**Histology, Immunofluorescence, and Fibrosis Quantification**

At the endpoint, hearts were arrested in diastole with 10% KCl, perfused with PBS, and fixed in 4% paraformaldehyde. The tissues were then paraffin-embedded and sectioned at a thickness of 5 µm. For fibrosis quantification, sections were stained with Picro-Sirius Red (Servicebio, GP1033)^[1]^. Images were captured using a digital slide scanner, and quantified using ImageJ software (NIH, ImageJ 1.54p with 64-bit Java 8, RRID:SCR_003070). Interstitial fibrosis was calculated in at least five different fields per heart as the percentage of the red-stained collagen area relative to the total myocardial tissue area, excluding large perivascular regions. Perivascular fibrosis was quantified separately by identifying several coronary arteries in cross-section for each heart. Using ImageJ, the area of collagen deposition immediately surrounding the vessel (adventitia) was measured and normalized to the vessel's luminal area to calculate the perivascular fibrosis ratio.

For immunofluorescence, deparaffinized sections underwent heat-induced antigen retrieval in a sodium citrate buffer. Sections were then blocked with a solution containing 5% donkey serum, 1% BSA, and 0.1% Triton-X100 before overnight incubation with primary antibodies (α-SMA, 1:500, CST, 19245) (CD45, 1:100, CST,70257S ) (CD68, 1:200, Bio-Rad, MCA1957) (CD31, 1:200, R&D, AF3628) (CD248, 1:100, Novus, MAB7535) (Postn, 1:300, Abcam, ab14041) (Vimentin, 1:250, Abcam, ab45939) at 4°C. Cell membranes were stained with Wheat Germ Agglutinin (10μg/ml, Invitrogen, W11262, Alexa Fluor 594-conjugated WGA) to measure myocyte cross-sectional area^[1]^. The cross-sectional area of at least 100 transversely cut cardiomyocytes per heart was measured using ImageJ. Capillary density was assessed by staining for *Griffonia Simplicifolia Lectin I* Isolectin B4 (1:300, Vector Laboratories, L-1104-1, GSL-I B4)^[1]^. Nuclei were counterstained with DAPI. Images were acquired with a confocal microscope (Olympus, FV3000). The number of capillaries and arterioles was counted and normalized to the tissue area to determine vessel density. Nuclei were counterstained with DAPI. All analysis was performed by an investigator blinded to the experimental groups.

**Cell Isolation, Culture, and *In Vitro* Assays**

Primary adult mouse cardiac mesenchymal cell were isolated from 8–12-week-old male C57BL/6J mice^[1]^. Ventricles were minced and subjected to enzymatic digestion with a solution containing collagenase type II (GIBCO, 17101015, 80 IU/mL) and trypsin (0.1%, ThermoFisher, 15090046) in DMEM. The resulting cell suspension was filtered, and fibroblasts were enriched by pre-plating for 3 hours, after which non-adherent cells were washed away. Enriched mCF were cultured in DMEM supplemented with 10% FBS and 1% Penicillin-Streptomycin. For knockdown experiments, cells were transfected with 30 nM of siRNA targeting *Cd248* series 1 (RNA#1: 5’-GCCUGCCCUAUAGGCCCAATT-3’; 5’-UUGGGCCUAUAGGGCAGGCTT-3’; RNA#2: 5’-CCUACCAACUCCAGGUCUUTT-3’; 5’-AAGACCUGGAGUUGGUAGGTT-3’; RNA#3: 5’-GCUGCUUGCCCUGGGCAUUTT-3’; 5’-AAUGCCCAGGGCAAGCAGCTT-3’) or series 2 (RNA#1: 5’-GCCUGCCCUAUAGGCCCAATT-3’; 5’-UUGGGCCUAUAGGGCAGGCTT-3’; RNA#2: 5’-CCUACCAACUCCAGGUCUUTT-3’; 5’-AAGACCUGGAGUUGGUAGGTT-3’; RNA#3: 5’-GCUGCUUGCCCUGGGCAUUTT-3’; 5’-AAUGCCCAGGGCAAGCAGCTT-3’) using Lipofectamine RNAiMAX (Invitrogen, 13778150) in Opti-MEM (GIBCO, 31985070). Myofibroblast activation was induced by treating cells with 5 ng/mL of recombinant TGF-β1 (ThermoFisher, 7666-MB-005) for 24 hours. For reverse knockdown experiments, cells were treated with 5 ng/mL of recombinant TGF-β1 for 8 hours followed by transfection with 30 nM of siRNA targeting *Cd248* for 48 hours.

Cell migration was assessed via a scratch-wound assay using a 200 µL pipette tip, with wound closure imaged at 0 and 24 hours. Cell proliferation was measured using a Click-iT EdU assay (Beyotime, C0078S), where cells were incubated with 10 µM EdU (Beyotime, C0078S) for 24 hours prior to fixation and detection.

**RNA Analysis and Transcriptome Sequencing**

Total RNA was extracted from cultured cells or tissues using RNAiso Plus reagent (TAKARA, 9019). For quantitative RT-PCR, 500-1000 ng of RNA was treated with gDNA Eraser and reverse-transcribed into cDNA using the PrimeScript RT Reagent Kit (TAKARA, RR047A). qPCR was performed using TB Green Premix Ex Taq II (TAKARA, RR820A) on a real-time PCR system. Relative gene expression was calculated using the ΔΔCt method. Primers are listed in Supplemental Table 1.

**Supplementary table 1**

| **qRT-PCR primers** | | |
| --- | --- | --- |
| **Gene** | **Forward Sequence** | **Reverse Sequence** |
| *Cd248* | TGAGTGTTACTGCAGCGAGG | GTGCCATCAAAGTCCTCCCA |
| *Acta2* | ACACGGCATCATCACCAACT | ATTTTCTCCCGGTTGGCCTT |
| *Col3a1* | ATTCTGCCACCCCGAACTC | TGTCCACCAGTGCTTACGTG |
| *Col1a1* | CCTGGCAAAGACGGACTCAA | GCTGAAGTCATAACCGCCAC |
| *Postn* | ATCAAGGTGCTATCTGCGGG | TCAATAGGCATCACTGCGGG |
| *Ninj1* | GTGGTCCTCATCTCTATCTCCC | CGACGATGATGAAAACCAGTCCC |
| *Akcr2* | GGCACTGCTATGCGGATTTTGG | AACGCAACCGATGCGGGAGTAA |
| *Akcr3* | GACCGCTATCTCTCCATCACCT | GTTGGAAGCAGATGTGACCGTC |
| *Sema3c* | CTGATAGTCCGCATAGGCACTG | GGCAGAGCTATTGGTAGGAAGG |
| *Dpp4* | CACCTCTGATGGAAGCAGCTTC | GATAATCGCTGGTCAGAGCTTCG |
| *Pgf* | TGCTGTGGTGATGAAGGTCTGC | GCATTCACAGAGCACATCCTGAG |
| *Fgf18* | CGAGATGATGTGAGTCGGAAGC | CCGAAGGTATCTGTCTCCACTAG |
| *S100a4* | AGCTCAAGGAGCTACTGACCAG | GCTGTCCAAGTTGCTCATCACC |
| *NbI1* | AGCCAAGAACATCACGCAGA | GTGTTGGGGACGCTGTAACT |

Total RNA of primary adult mouse cardiac mesenchymal cells prepared as described above was subjected to transcriptome sequencing on DNBSEQ platform (BGI Genomics) following the manufacturer’s instruction, resulting in ~44 M raw reads each sample. Raw reads were assessed for quality, filtered out of adapter content, reads containing redundant unknown bases and below Q20 using SOAPnuke v1.5.2 software (https://github.com/BGI-flexlab/SOAPnuke). Clean reads filtered from raw reads were aligned to mouse genome (NCBI, Mus musculus, GCF_000001635.27_GRCm39) using HISAT and Bowtie2. Differentially expressed genes (DEG) applied were analyzed using DESeq2 and identified as significant DEG with a Q-Value ≤ 0.05.

**Single-Cell RNA-seq Analysis**

The public mouse heart single-cell RNA sequencing dataset GSE166403^[5]^ CRA022616, and CRA005739 was downloaded from NCBI and re-analyzed using the Seurat package (RRID:SCR_007322) in R. Cells with fewer than 200 or more than the 95th percentile of features, or with mitochondrial gene content >20%, were excluded. Data were normalized, and datasets were integrated using Harmony. Dimensionality was reduced using PCA, and cells were clustered and visualized with UMAP.

**single-cell RNA-seq data and single-nucleus RNA-seq analysis**

Analysis of single-cell RNA-seq (scRNA-seq) and single-nucleus RNA-seq (snRNA-seq) data was performed using R v4.5.1 (https://www.r-project.org/) including several R packages which were listed in Supplemental table 2.

Data acquisition and filtering

scRNA-seq data GSE166403 was downloaded from NCBI (https://www.ncbi.nlm.nih.gov/). snRNA-seq data was downloaded from https://doi.org/10.6084/m9.figshare.c.5777948.v2^[6]^. First, the expression matrix of scRNA-seq and snRNA-seq was imported into Seurat v5.3.0 to create Seurat objects. For all samples of scRNA-seq, we filtered cells with less than 500 features, more than 5000 features, or more than 25 mitochondrial gene counts. Then we merged all the filtered samples from day 0 and day 14 into one Seurat object. The samples of snRNA-seq were already filtered before being downloaded, and we merged all of them into another Seurat object.

Processing of the scRNA-seq and snRNA-seq data

The merged scRNA-seq data and snRNA-seq data were processed using the standard Seurat workflow respectively. The merged data were normalized with the “LogNormalize” method (scale factor = 10,000), and the top 2000 variable features were identified using the “vst” method. The data was scaled and subjected to PCA with 50 components. Batch effects across samples were corrected using Harmony v1.2.3 on the first 30 PCs. A shared nearest neighbor graph was then constructed on the Harmony-reduced space (30 dimensions), followed by clustering with the Louvain algorithm (resolution = 0.5). Finally, UMAP was performed on the Harmony embeddings (30 dimensions) for visualization.

Annotation

For both scRNA-seq and snRNA-seq data, marker genes of each cluster were calculated by the Seurat function FindAllMarkers. Cluster annotation was performed based on canonical marker genes curated from CellMarker 2.0 (http://bio-bigdata.hrbmu.edu.cn/CellMarker) (RRID:SCR_018503) and the original publication of the dataset used in this study. Clusters were renamed accordingly using the RenameIdents function in Seurat. Expression patterns of marker genes across clusters were visualized with the Seurat function DotPlot.

*Cd248* and *Postn* expression

The spatial distribution and co-expression of *Cd248* and *Postn* were visualized using the “FeaturePlot” function with the blending option enabled. The proportions of *Cd248*⁺, *Postn*⁺, and double-positive cells were quantified within each cluster and sample group based on expression values (>0.5) extracted by “FetchData,” and the results were displayed as grouped bar plots with ggplot2 (RRID:SCR_014601).

To further examine fibroblast subpopulations in the TAC sample, cells were classified into four groups according to expression thresholds of *Cd248* > 0.5 and *Postn* > 0.5 (*Cd248⁺Postn⁻*, *Cd248⁻Postn⁺*, *Cd248⁺Postn⁺*, *Cd248⁻Postn⁻*), and their distribution was projected onto UMAP embeddings using the DimPlot function.

DEGs analysis

Differentially expressed genes (DEGs) were identified in fibroblasts of the TAC sample using the Seurat function FindMarkers (min.pct = 0.1). Comparisons were performed between *Postn*⁺*Cd248*⁺ and *Postn*⁻*Cd248*⁺ fibroblasts, as well as between *Postn*⁺*Cd248*⁺ and *Postn*⁺*Cd248*⁻ fibroblasts. Volcano plots were generated using the ggplot2 package, where significantly differentially expressed genes were defined as those with an absolute log₂ fold change > 1 and an adjusted *p*-value < 0.05.

GO analysis
Differentially expressed genes were mapped to Entrez IDs using the bitr function in clusterProfiler (RRID:SCR_016884). GO enrichment was then performed with enrichGO (ontology = “ALL”, adjusted *p*-value < 0.05), and results were visualized with dotplot, separating biological process (BP), molecular function (MF), and cellular component (CC) categories.

**supplementary table 2**

| attached packages and version | loaded via a namespace (+version) |
| --- | --- |
| Seurat(5.3.0) | SeuratObject(5.2.0) |
| harmony(1.2.3) | sp(2.2-0) |
| ggplot2(4.0.0) | Rccp(1.1.0) |
| ggrepel(0.9.6) | AnnotationDbi(1.70.0) |
| readxl(1.4.5) | base(4.5.1) |
| readr(2.1.5) | Bioconductor(0.54.0) |
| dyplr(1.1.4) | datasets(4.5.1) |
| clusterProfiler(4.16.0) | generics(0.1.4) |
| org.Mm.eg.db(3.21.0) | graphics(4.5.1) |
| tidyr(1.3.1) | grDevices(4.5.1) |
|  | methods(4.5.1) |
|  | S4Vectors(0.46.0) |
|  | stats(4.5.1) |
|  | stats4(4.5.1) |
|  | utils(4.5.1) |

**Statistical Analysis**

All quantitative data are presented as mean ± SEM. Statistical analyses were performed using GraphPad Prism software (v9.0, GraphPad Software, RRID:SCR_002798). The normality of data distribution was assessed using the Shapiro-Wilk test to determine the appropriate statistical approach. For comparisons between two groups with normally distributed data, an unpaired, two-tailed Student’s t-test was used. For comparisons between more than two groups with normally distributed data, a one-way or two-way Analysis of Variance (ANOVA) was performed, followed by Tukey’s multiple comparisons post-hoc test. For data that were not normally distributed, the non-parametric Mann-Whitney U test (for two groups) or the Kruskal-Wallis test with Dunn's multiple comparisons test (for multiple groups) was used. Survival curves were generated using the Kaplan-Meier method and compared using the Log-rank (Mantel-Cox) test. A P-value < 0.05 was considered statistically significant. The specific tests used for each experiment are detailed in figure legends.

**Reference**

[1] XIANG F-L, FANG M, YUTZEY K E. Loss of β-catenin in resident cardiac fibroblasts attenuates fibrosis induced by pressure overload in mice [J]. Nat Commun, 2017, 8(1): 712.

[2] KHALIL H, KANISICAK O, PRASAD V, et al. Fibroblast-specific TGF-β-Smad2/3 signaling underlies cardiac fibrosis [J]. J Clin Invest, 2017, 127(10): 3770-83.

[3] XIANG F-L, GUO M, YUTZEY K E. Overexpression of Tbx20 in Adult Cardiomyocytes Promotes Proliferation and Improves Cardiac Function After Myocardial Infarction [J]. Circulation, 2016, 133(11): 1081-92.

[4] LI J, XIAO F, LIN B, et al. Ferrostatin-1 improves acute sepsis-induced cardiomyopathy via inhibiting neutrophil infiltration through impaired chemokine axis [J]. Front Cell Dev Biol, 2024, 12: 1510232.

[5] PEISKER F, HALDER M, NAGAI J, et al. Mapping the cardiac vascular niche in heart failure [J]. Nat Commun, 2022, 13(1): 3027.

[6] LIU X, YIN K, CHEN L, et al. Lineage-specific regulatory changes in hypertrophic cardiomyopathy unraveled by single-nucleus RNA-seq and spatial transcriptomics [J]. Cell Discov, 2023, 9(1): 6.
